# Direct conversion of pig fibroblasts to chondrocyte-like cells by c-Myc

**DOI:** 10.1038/s41420-018-0136-4

**Published:** 2019-01-18

**Authors:** Jun-Wen Shi, Ting-Ting Zhang, Wei Liu, Jie Yang, Xiao-Lin Lin, Jun-Shuang Jia, Hong-Fen Shen, Sheng-Chun Wang, Jing Li, Wen-Tao Zhao, Wei-Wang Gu, Yan Sun, Dong Xiao

**Affiliations:** 1 Guangdong Provincial Key Laboratory of Cancer Immunotherapy Research and Guangzhou Key Laboratory of Tumor Immunology Research, Cancer Research Institute, Southern Medical University, Guangzhou, 510515 China; 2 Institute of Comparative Medicine & Laboratory Animal Center, Southern Medical University, Guangzhou, 510515 China; 3 Department of Oncology, the First People’s Hospital of Chenzhou, Chenzhou, 423000 China; 4 Zhongshan School of Medicine, Sun Yat-sen University, Guangzhou, 510080 China

## Abstract

Unexpectedly, we found that c-Myc-expressing porcine embryonic fibroblasts (PEFs) subcutaneously implanted into nude mice formed cartilage-like tissues in vivo, while previous studies revealed the direct conversion of mouse and human somatic cells into chondrocytes by the combined use of several defined factors, including c-Myc, which prompted us to explore whether PEFs can be reprogrammed to become pig induced chondrocyte-like cells (piCLCs) via ectopic expression of c-Myc alone. In this study, c-Myc-expressing PEFs, designated piCLCs, which exhibited a significantly enhanced proliferation ability in vitro, displayed a chondrogenic phenotypes in vitro, as shown by the cell morphology, toluidine blue staining, alcian blue staining and chondrocyte marker gene expression. Additionally, piCLCs with a polygonal chondrocyte-like morphology were readily and efficiently converted from PEFs by enforced c-Myc expression within 10 days, while piCLCs maintained the chondrocytic phenotype and normal karyotype during long-term subculture. piCLC-derived single clones with a chondrogenic phenotype in vitro exhibited homogeneity in cell morphology and staining intensity compared with mixed piCLCs. Although the mixtures of cartilaginous tissues and tumorous tissues accounted for ~12% (6/51) of all xenografts (51), piCLCs generated stable, homogenous, hyaline cartilage-like tissues without tumour formation at 45 out of the 51 injected sites when subcutaneously injected into nude mice. The hyaline cartilage-like tissues remained for at least 16 weeks. Taken together, these findings demonstrate for the first time the direct induction of chondrocyte-like cells from PEFs with only c-Myc.

## Introduction

c-Myc belongs to the Myc family of transcription factors, which also includes N-Myc and L-Myc. c-Myc is believed to regulate the expression of 15% of all genes. By modifying the expression of its target genes, c-Myc plays important roles in the control of normal cell proliferation, growth, differentiation, apoptosis, survival, stem cell self-renewal, establishment and maintenance of pluripotency, and other processes^1–8^.

Our previous study is first to reveal that the enforced expression of c-Myc in porcine embryonic fibroblasts (PEFs) triggered epithelial-like morphological conversion and mesenchymal-epithelial transition (MET) via F-actin reorganization and RhoA/Rock pathway inactivation^9^. In our pilot experiment, we unexpectedly found that c-Myc-expressing PEFs generated cartilage-like tissues when subcutaneously injected into nude mice. Mouse and human somatic cells (including fibroblasts) can be directly converted into chondrocyte-like cells by a different set of transcription factors, including the combined transduction of two reprogramming factors (c-Myc and Klf4) and one chondrogenic factor (SOX9)^10,11^, and a combination of only five genes (5 F pool)—c-Myc, BCL6, T, MITF and BAF60C^12^, indicating that the aforementioned two reprogramming systems for directly inducing chondrocytes from various somatic cells share a common gene, c-Myc. Moreover, c-Myc is a critical reprogramming factor for induced pluripotent stem cells (iPS cells or iPSCs) reprogrammed from animal and human somatic cells (including fibroblasts) by defined factors (Oct4, Sox2, c-Myc and Klf4)^13,14^. Furthermore, increasing evidence demonstrates that the proto-oncogene c-Myc is also involved in chondrocyte proliferation, differentiation and maturation, as well as bone formation (see Discussion for details)^15–21^. These above-mentioned findings prompted us to suspect that PEFs can be directly converted into pig induced chondrocyte-like cells (piCLCs) by only c-Myc, which has never been reported. In the present study, we examined whether piCLCs could be directly induced from PEFs pig fibroblasts by the re-expression of c-Myc alone.

## Results

### Ectopic expression of c-Myc in PEFs improves cell proliferation capacity

**Fig. 1:**
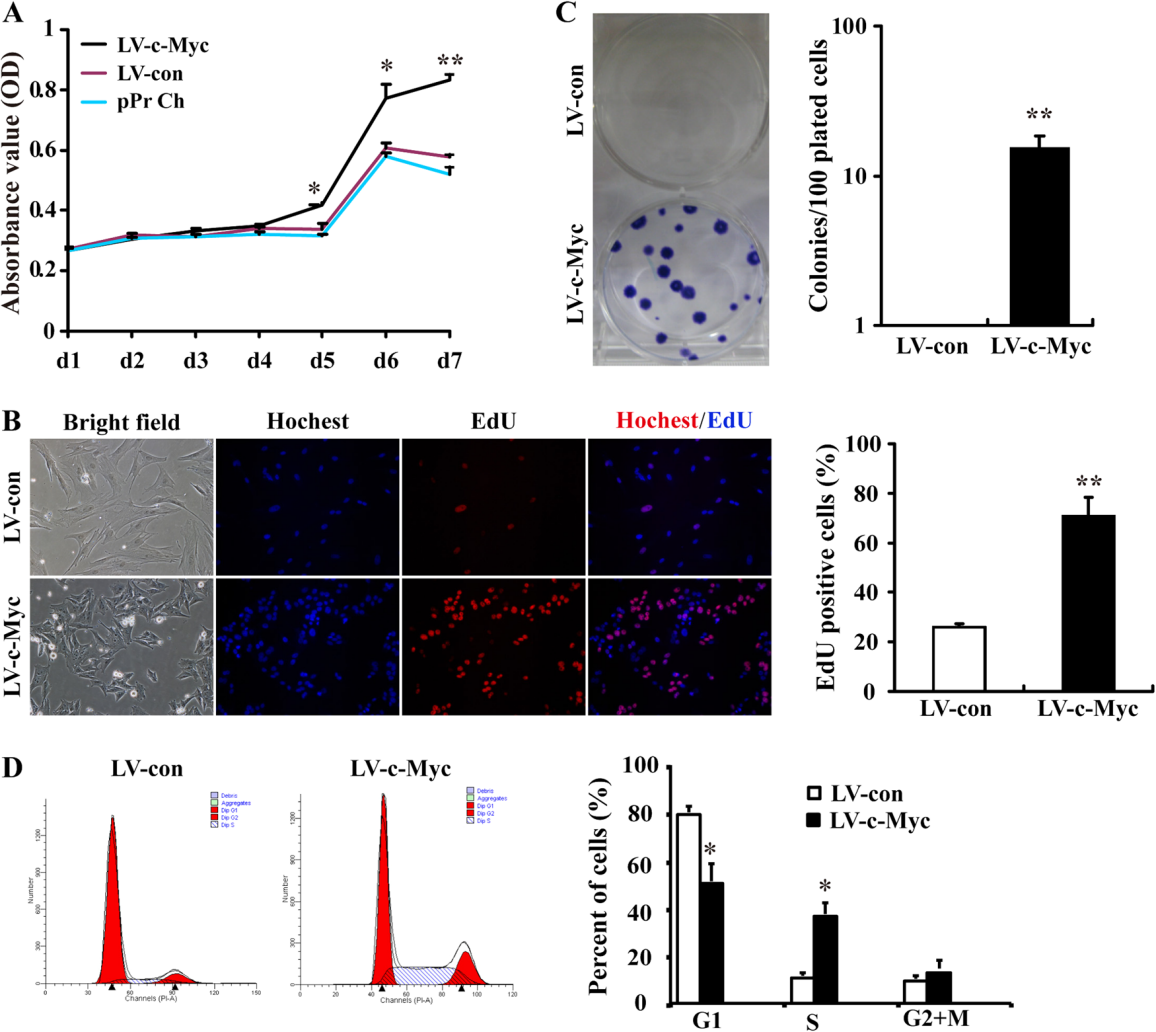
c-Myc-expressing PEFs display enhanced proliferation ability. **a** The proliferation ability of c-Myc-expressing PEFs (LV-c-Myc), vector-expressing PEFs (LV-con) and pPr Ch was analysed by CCK8 assay. **b** Colony formation assay for c-Myc-expressing PEFs. **c** EdU assay for the proliferation ability of c-Myc-expressing PEFs. **d** Representative histograms for cell-cycle distribution of c-Myc-expressing PEFs

Our findings described in 'Supplementary Results' section demonstrate that c-Myc-expressing PEFs, which underwent an epithelial-like morphological change and MET induced by the enforced c-Myc expression^9^, displayed enhanced proliferation ability in vitro compared with vector-expressing PEFs (Fig. 1; Fig. S1 and S2, Supplementary information).

### In vivo cartilage-like tissue formation by c-Myc-expressing PEFs in nude mice

**Fig. 2:**
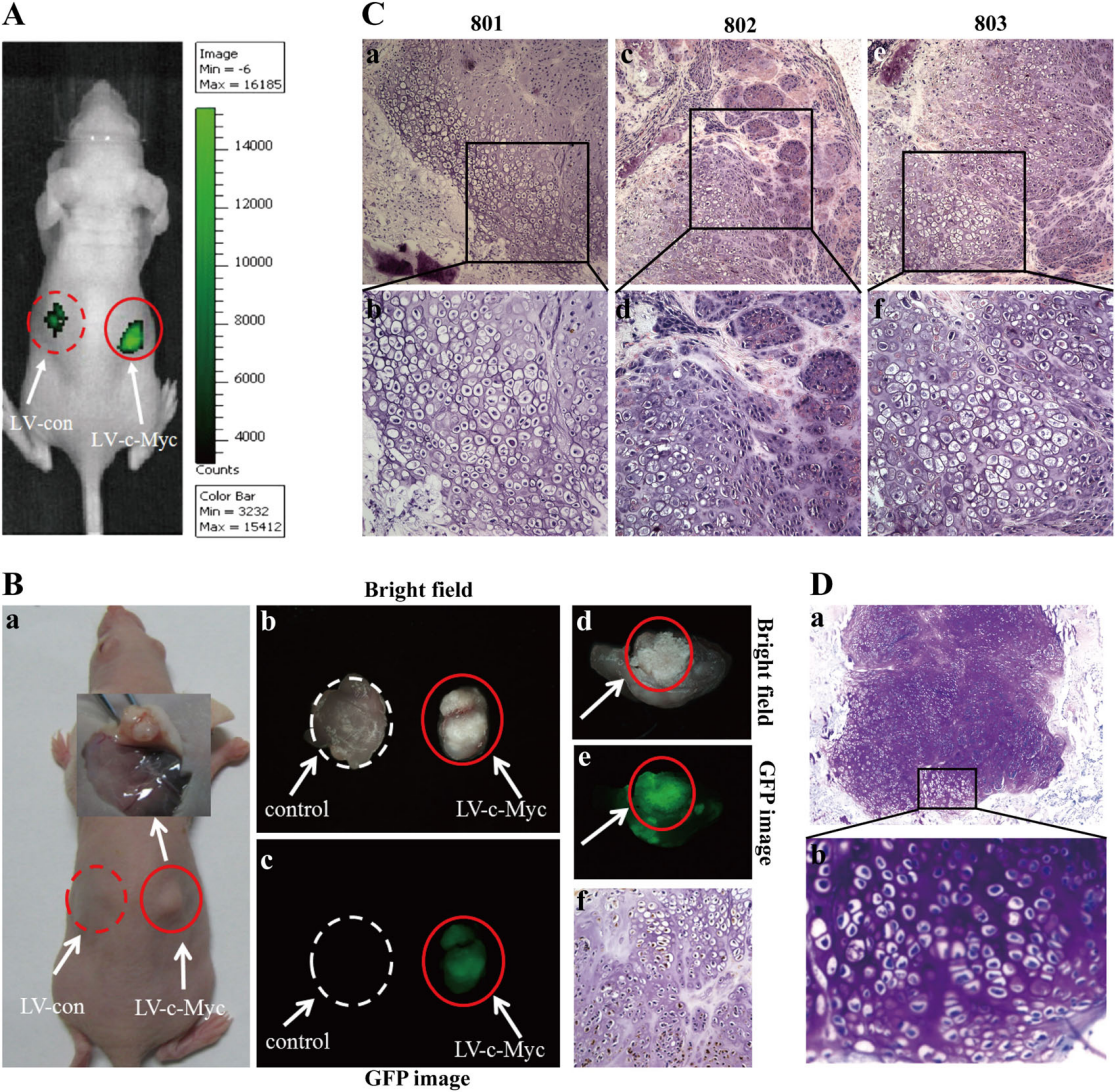
In vivo cartilage-like tissue formation by c-Myc-expressing PEFs. Vector and c-Myc-expressing PEFs were injected subcutaneously into the dorsal flank of nude mice (*n* = 5). The xenografts became palpable at sites injected with c-Myc-expressing PEFs 2 weeks after inoculation, and three out of five mice developed xenografts at the end of the experiment, whereas no xenografts were observed at sites injected with vector-expressing PEFs. **a** In vivo EGFP assay for the transplanted EGFP-positive vector-expressing PEFs (left) and c-Myc-expressing PEFs (right) at injected sites of nude mice by the IVIS Lumina Imaging System. **b** The suspected cartilage-like tissues developed from the transplanted c-Myc-expressing PEFs at injected sites (right) of a nude mouse (801). **B**-**a** Picture of a nude mouse (801) [shown in (A)] xenograft. **B**-**b,c** Assay of GFP expression in the xenograft stripped from a nude mouse (801) under stereo fluorescence microscope. Control tissues were obtained from the same nude mouse (801). **B**-**d** Picture of the cross section of GFP-positive graft harbouring LV-c-Myc [shown in (**B**-**b**,**c**; right)]. **B**-**e** GFP assay for the cross-section [shown in (**B**-**d**)] of a GFP-positive graft. **B**-**f** GFP-stained section of transplanted xenograft tissue formed by c-Myc-expressing PEFs. **c** HE staining of the suspected cartilage-like tissues formed from the transplanted c-Myc-expressing PEFs at injected sites of 3 nude mice (801, 802 and 803). Images (**b**, **d** and **f**) are higher magnifications of the rectangular regions indicated in images (**a**, **c** and **e**), respectively. Original magnification: 40×. **d** Toluidine blue staining of tissue sections shown in (**c**). **b** is a higher magnification of the rectangular region indicated in (**a**)

Following the observation of c-Myc–mediated in vitro growth promotion (Fig. 1) in c-Myc-expressing PEFs undergoing c-Myc-induced MET^9^, vector and c-Myc-expressing PEFs were injected subcutaneously into the dorsal flank of nude mice to further explore the influence of c-Myc on PEF growth in vivo (Fig. 2a). The xenografts became palpable at sites injected with c-Myc-expressing PEFs 2 weeks after inoculation, and three of five mice developed xenografts at the end of the experiment, whereas no xenografts were observed at sites injected with vector-expressing PEFs (Fig. 2B-a). Four weeks after injection, we sacrificed the mice and grossly and histologically examined the injected sites. Unlike tumour xenografts formed subcutaneously in nude mice, we found, unexpectedly, that the xenograft peeled from a nude mouse (#801) had a relatively hard texture and displayed white colour (shown in Fig. 2B-b, right). When the peeled xenograft (shown in Fig. 2B-b, right) was cut in half by a surgical blade, gross examination showed that the cross-section of xenograft was consisted largely of the suspected cartilage-like tissues (Fig. 2B-d and Supplementary information, Fig. S3). The same results obtained from nude mouse (#801) (Fig. 2B-d and Fig. S3, Supplementary information) were also observed in nude mice #802 and #803 (data not shown). More importantly, histological examination by HE staining (Fig. 2c) and toluidine blue staining (Fig. 2d) revealed that pig xenografts consisted largely of the substantial amounts of homogenous cartilage tissues, as indicated by metachromatic staining with toluidine blue and lacuna formation, which are typical of cartilage. The xenograft (shown in Fig. 2B-b, right) dissected from nude mouse #801 displayed GFP fluorescence (Fig. 2B-c, e), and the sections of transplanted xenograft tissues positively stained for GFP (Fig. 2B-f), indicating that the xenograft was developed from the transplanted GFP-positive c-Myc-expressing PEFs. On the other hand, histological examination by HE staining showed that injection of c-Myc-expressing PEFs did not produce tumours at injected sites of nude mice #801, 802 and 803 within 4 weeks after injection (data not shown). These results show that c-Myc-expressing PEFs subcutaneously implanted into nude mice formed cartilage-like tissues in vivo.

### piCLCs from PEFs by the ectopic c-Myc expression display chondrocyte characteristics

As mentioned in the Introduction, mouse and human somatic cells (including fibroblasts) can be directly reprogrammed into chondrocyte-like cells by defined factors^10–12^. In this study, we found that c-Myc-expressing PEFs generated cartilage-like tissues when subcutaneously injected into nude mice (Fig. 2). Based on the above-mentioned findings from other labs and this study, we hypothesized that PEFs can be directly converted into piCLCs by c-Myc alone. Subsequently, we wanted to define whether c-Myc-expressing PEFs displayed chondrocyte-like phenotypes.

Our findings described in 'Supplementary Results' section show that the methods for isolating, culturing and identifying the chondrocytes of mice and pigs were successfully established in our lab (Fig. S1, Fig. S2, Supplementary information).

**Fig. 3:**
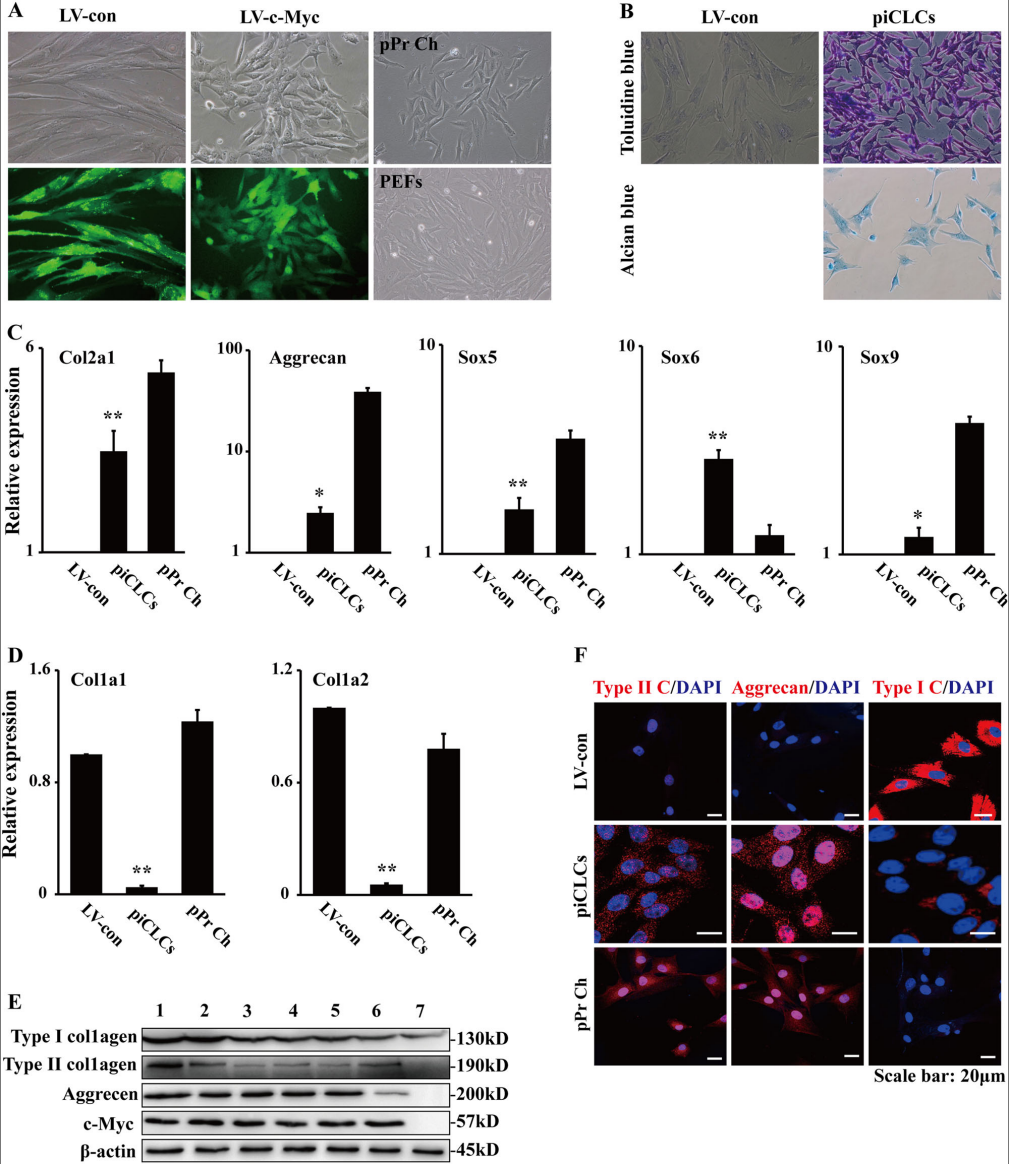
Chondrocyte marker gene expression analyses of pig induced chondrocyte-like cells (piCLCs) from PEFs by c-Myc. **a** Cell morphologies of PEFs, pPr Ch and piCLCs. The phase-contrast photographs of c-Myc-expressing PEFs (i.e., piCLCs) and vector-expressing PEFs were token at day 4 post-infection. pPr Ch: porcine primary chondrocytes. **b** Toluidine blue staining and alcian blue staining for piCLCs. Proteoglycan was identified by toluidine blue staining and alcian blue staining. **c**, **d** qRT-PCR analysis of chondrocyte (**c**) and fibroblast (**d**) marker gene expression in the indicated cells. In (**c**) and (**d**), error bars indicate mean ± SD (*n* = 3). **e** Cell extracts from pPr Ch, piCLCs and PEFs were analysed by immunoblotting with antibodies against the indicated proteins. Lane 1: pPr Ch; Lane 2: piCLCs P7; Lane 3: piCLCs P14; Lane 4: piCLCs P19; Lane 5: piCLCs P25; Lane 6: piCLCs-S; Lane 7: PEFs. **f** Immunofluorescence analysis showing protein expression of type I collagen (type I C), type II collagen (type II C) and aggrecan in the indicated cells. Nuclei were counterstained with DAPI

Our previous study was the first to demonstrate that c-Myc-expressing PEFs undergo MET to exhibit epithelial-like morphological changes^9^. The cell morphology of c-Myc-expressing PEFs was quite similar to the cell shape of the cultured pPr Ch, whereas there was a great difference in cell morphology between c-Myc-expressing PEFs and morphologically fibroblastic cells (i.e., vector-expressing PEFs or PEFs) (Fig. 3a and Supplementary information, Fig. S1). To detect glycosaminoglycan production in c-Myc-expressing PEFs (i.e., piCLCs), we stained dishes with toluidine blue or alcian blue. As shown in Fig. 3b, almost 100% of c-Myc-expressing PEFs were specifically and intensely stained with toluidine blue, indicating the existence of acid glycosaminoglycans, which is an element of the cartilage extracellular matrix. Therefore, the efficiency of the induction of toluidine blue-positive cells was almost 100%. On the other hand, the transduction of PEFs with LV-con did not result in substantial toluidine blue staining (Fig. 3b). Additionally, these c-Myc-expressing PEFs undergoing epithelial-like morphological conversion were also specifically and intensely stained with alcian blue (Fig. 3b). qRT-PCR analysis illustrated that c-Myc-expressing PEFs expressed chondrocyte marker genes, such as type II collagen alpha 1 chain (Col2a1), aggrecan, Sox5, Sox6 and Sox9, whereas the parental vector-expressing PEFs did not (Fig. 3c). On the other hand, qRT-PCR showed that c-Myc-expressing PEFs hardly expressed both fibroblast-associated type I collagen alpha 1 chain gene (Col1a1) and type I collagen alpha 2 chain gene (Col1a2), whereas the parental vector-expressing PEFs expressed these genes at high levels (Fig. 3d). Compared to the control, the cultured pPr Ch expressed Col1a1 and Col1a2 (Fig. 3d), probably due to minor contamination of fibroblasts during the harvesting procedure or de-differentiation of chondrocytes. Western blot analysis revealed that cultured pPr Ch and c-Myc-expressing PEFs expressed type II collagen, aggrecan and c-Myc, whereas the parental PEFs did not (Fig. 3e). Furthermore, immunofluorescence staining showed that c-Myc-expressing PEFs expressed type II collagen and aggrecan (Fig. 3f), which was consistent with the results from qRT-PCR (Fig. 3c) and Western blot (Fig. 3e). A few c-Myc-expressing PEFs weakly expressed type I collagen (Fig. 3f), which was similar to the results from qRT-PCR (Fig. 3d) and Western blot (Fig. 3e). In the cultured pPr Ch samples, all cells expressed type II collagen and aggrecan, and none of the cells expressed type I collagen (Fig. 3f). Moreover, vector-expressing PEFs expressed type I collagen, but not type II collagen or aggrecan, in culture (Fig. 3f). Together, these results suggest that the forced expression of c-Myc alone can directly reprogram PEFs into piCLCs in vitro.

### Reprogramming processes of piCLCs from PEFs by the ectopic c-Myc expression

**Fig. 4:**
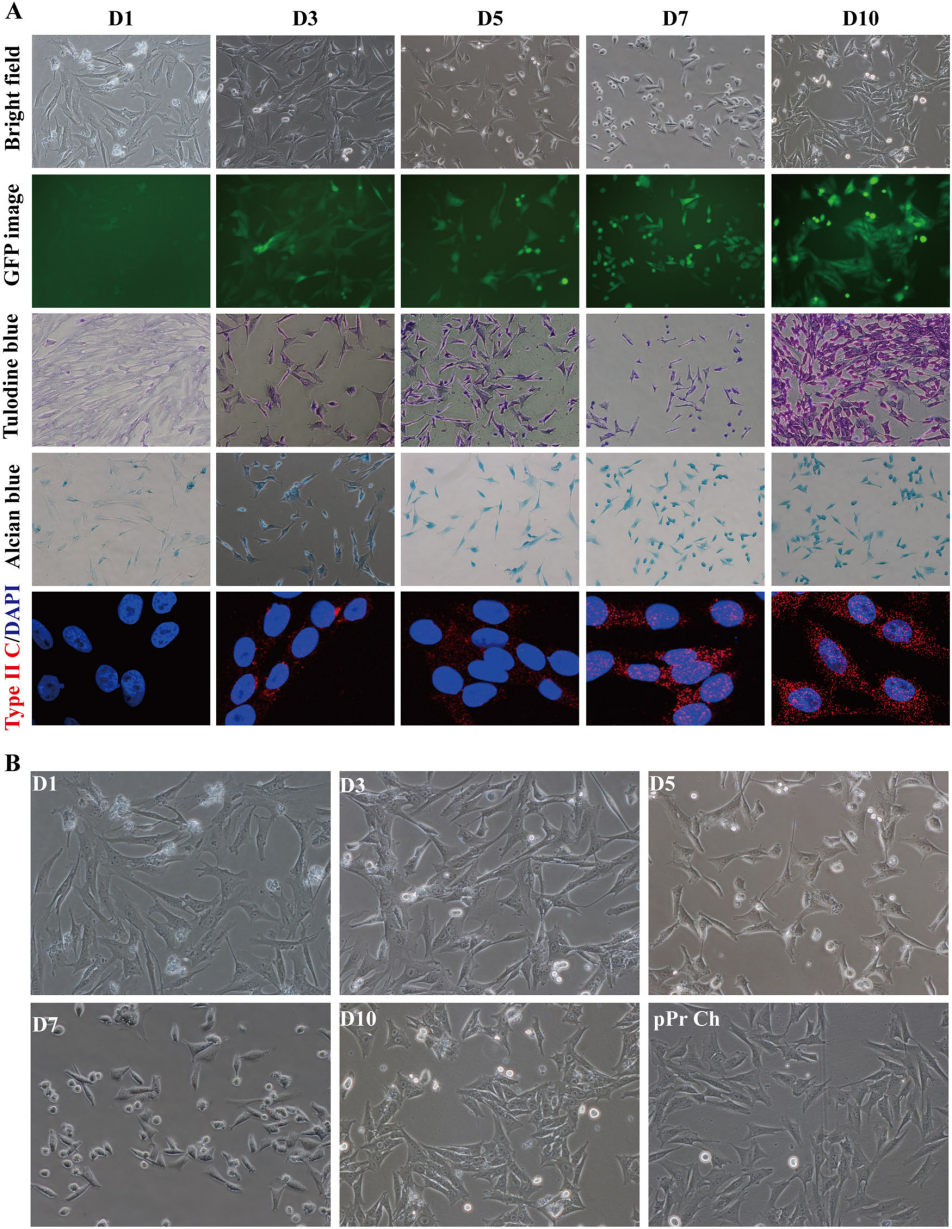
Reprogramming processes of piCLCs from PEFs by the ectopic c-Myc expression. **a** Reprogramming processes displayed by cell morphological changes, special staining and type II collagen (type II C) expression. **b** Cell morphological changes of c-Myc-expressing PEFs from D1 to D10

After the chondrocyte characteristics of piCLCs were confirmed in vivo and in vitro (Fig. 2 and Fig. 3), we dynamically monitored the reprogramming processes of piCLCs from PEFs by only c-Myc at 1 (D1), 3 (D3), 5 (D5), 7 (D7) and 10 days (D10) after transduction, as displayed by cell morphological changes, toluidine blue staining, alcian blue staining and type II collagen expression. Our results revealed that 3 days after lentiviral transduction, c-Myc-expressing PEFs began to change gradually from a long spindle shape to a short spindle shape or polygonal shape during the induction process (Figs. 4a, b). Five days after transduction, the morphology of c-Myc-expressing PEFs (Figs. 4a, b) was completely different from that of parental PEFs (Fig. S1B, Supplementary information) and vector-expressing PEFs (Fig. S5, Supplementary information). Toluidine blue and alcian blue staining revealed that c-Myc-expressing PEFs were weakly stained with cationic dyes (such as toluidine blue and alcian blue) on D3 and moderately stained with cationic dyes on D5 and D7 (Fig. 4a). As shown in Figs. 4a, b, 10 days after infection, c-Myc-expressing PEFs (i.e., piCLCs) exhibited the typical short spindle-like or polygonal-like morphology of cultured pPr Ch (Fig. S1B and Fig. 4b, Supplementary information), with intense toluidine blue and alcian blue staining compared with vector-expressing PEFs (Fig. S5, Supplementary information), which had a fibroblast-like appearance, indicating proteoglycan production in induced cells. Immunofluorescence staining demonstrated that by D3 and by D5, almost all c-Myc-expressing PEFs weakly or moderately expressed type II collagen, while by D7 and by D10, all c-Myc-expressing PEFs showed strong type II collagen expression (Fig. 4a), which was consistent with the results from toluidine blue and alcian blue staining (Fig. 4a). On the other hand, vector-expressing PEFs did not display these changes that were observed in c-Myc-expressing PEFs (Fig. 4 and Fig. S5, Supplementary information). Taken together, these results suggest that piCLCs with short spindle-like or polygonal-like morphology can be readily and efficiently converted from PEFs by ectopic c-Myc expression within 10 days.

### Chondrocyte marker gene expression analyses of piCLCs-S

**Fig. 5:**
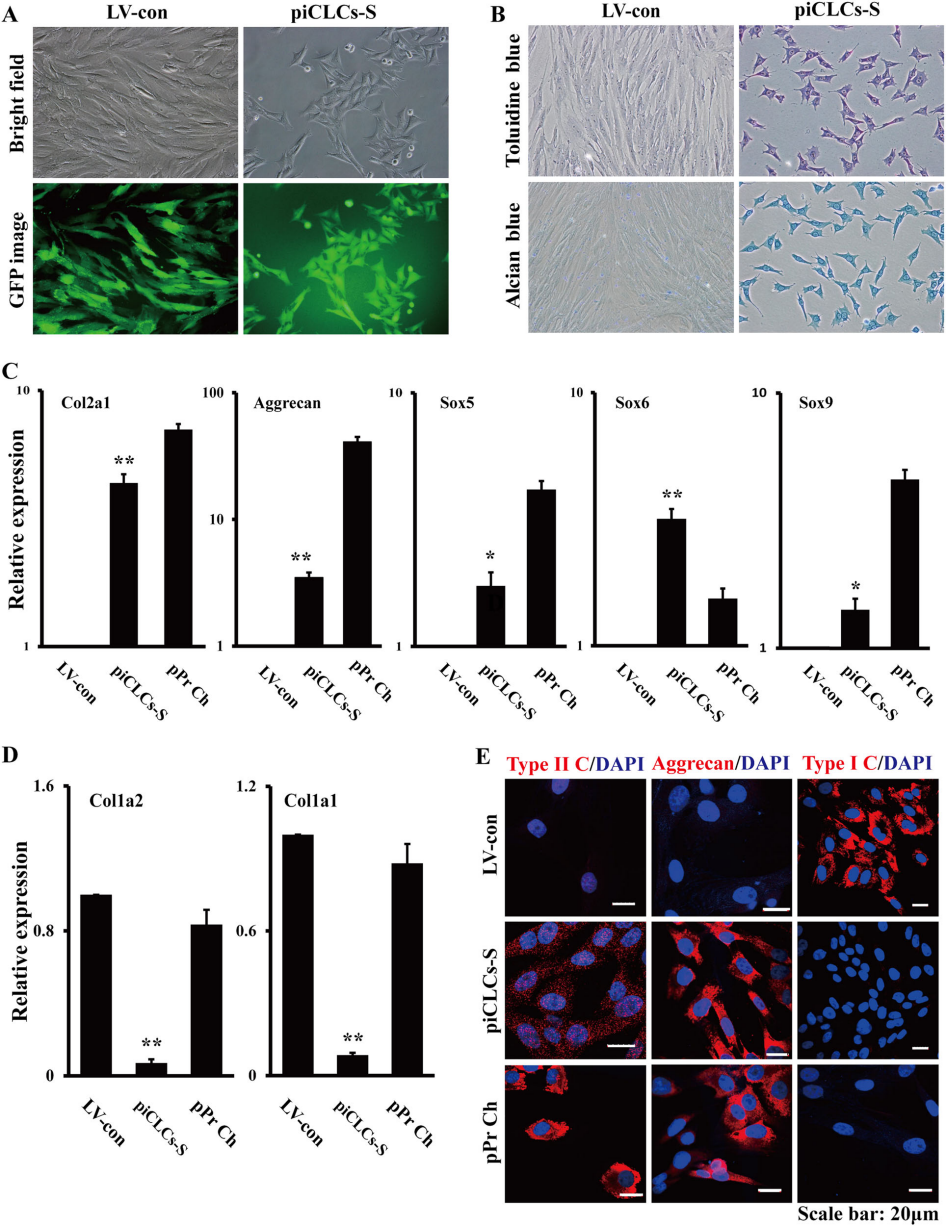
Chondrocyte marker gene expression analyses of the single subclone derived from mixed piCLCs (referred to as piCLCs-S). **a** Cell morphologies of piCLCs-S. **b** Toluidine blue staining and alcian blue staining of piCLCs-S. **c, d** qRT-PCR analysis of chondrocyte (**c**) and fibroblast (**d**) marker gene expression in the indicated cells. In (**c**) and (**d**), error bars indicate mean ± SD (*n* = 3). **e** Immunofluorescence analysis showing protein expression of type I collagen (type I C), type II collagen (type II C) and aggrecan in the indicated cells. Nuclei were counterstained with DAPI

As shown in Fig. 3 and Fig. 4, almost 100% of mixed piCLCs with the chondrogenic phenotype displayed metachromatic toluidine blue and alcian blue staining with different staining intensities, suggesting the heterogeneity of mixed piCLC populations. To investigate whether single clones derived from mixed piCLCs (hereinafter referred to as piCLCs-S) exhibited a chondrogenic phenotype in vitro, the limiting dilution technique was employed to isolate single clones. Our findings described in 'Supplementary Results' section demonstrate that piCLCs-S with a chondrogenic phenotype in vitro exhibits homogeneity in cell morphology and staining intensity compared with mixed piCLCs (Fig. 5).

### Chondrocyte phenotypic stability assay, tumourigenicity assay and karyotype analysis for piCLCs

**Fig. 6:**
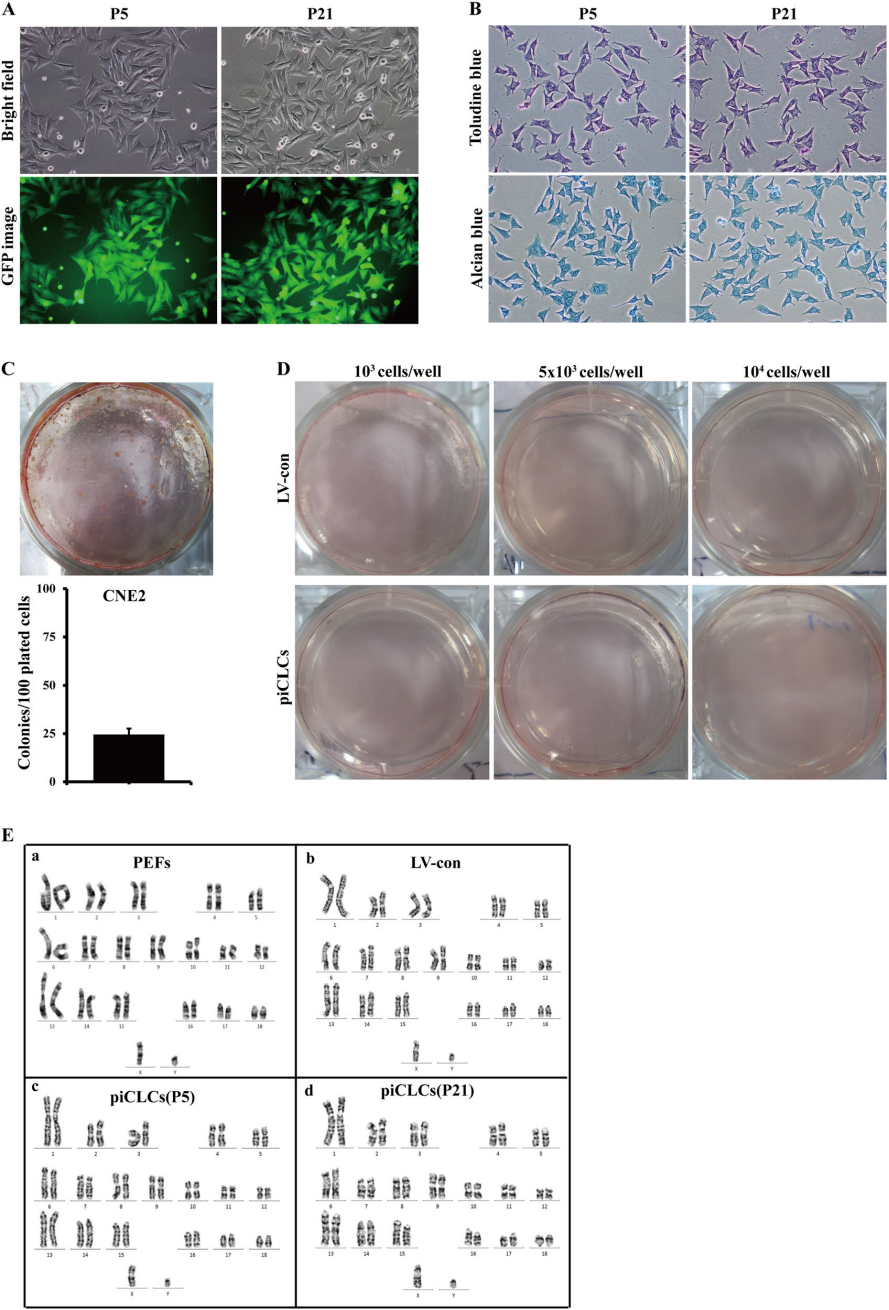
Chondrocyte phenotypic stability assay, tumourigenicity assay and karyotype analysis for piCLCs. **a** Cell morphologies of passage 5 (P5) and passage 21 (P21) piCLCs. **b** Toluidine blue staining and alcian blue staining for early P5 and late P21 piCLCs. **c**, **d** Soft-agar colony formation assay for CNE2 cells (**c**) and piCLCs (**d**). **c** Soft-agar colony formation assay for CNE2 cells. CNE2 cells, a nasopharyngeal carcinoma cell line, were used as a positive control. **d** Soft-agar colony formation assay for piCLCs (10^3^ cells/well, 5 × 10^3^ cells/well and 10^4^ cells/well). **e** Karyotype analysis for P5 and P21 piCLCs

Our findings described in 'Supplementary Results' section demonstrate that piCLCs maintained the chondrocytic phenotype and normal karyotype during long-term subculture, and had no capacity for anchorage-independent growth in vitro (Fig. 6).

### Prolonged hyaline cartilage-like tissue formation by piCLCs in nude mice

**Fig. 7:**
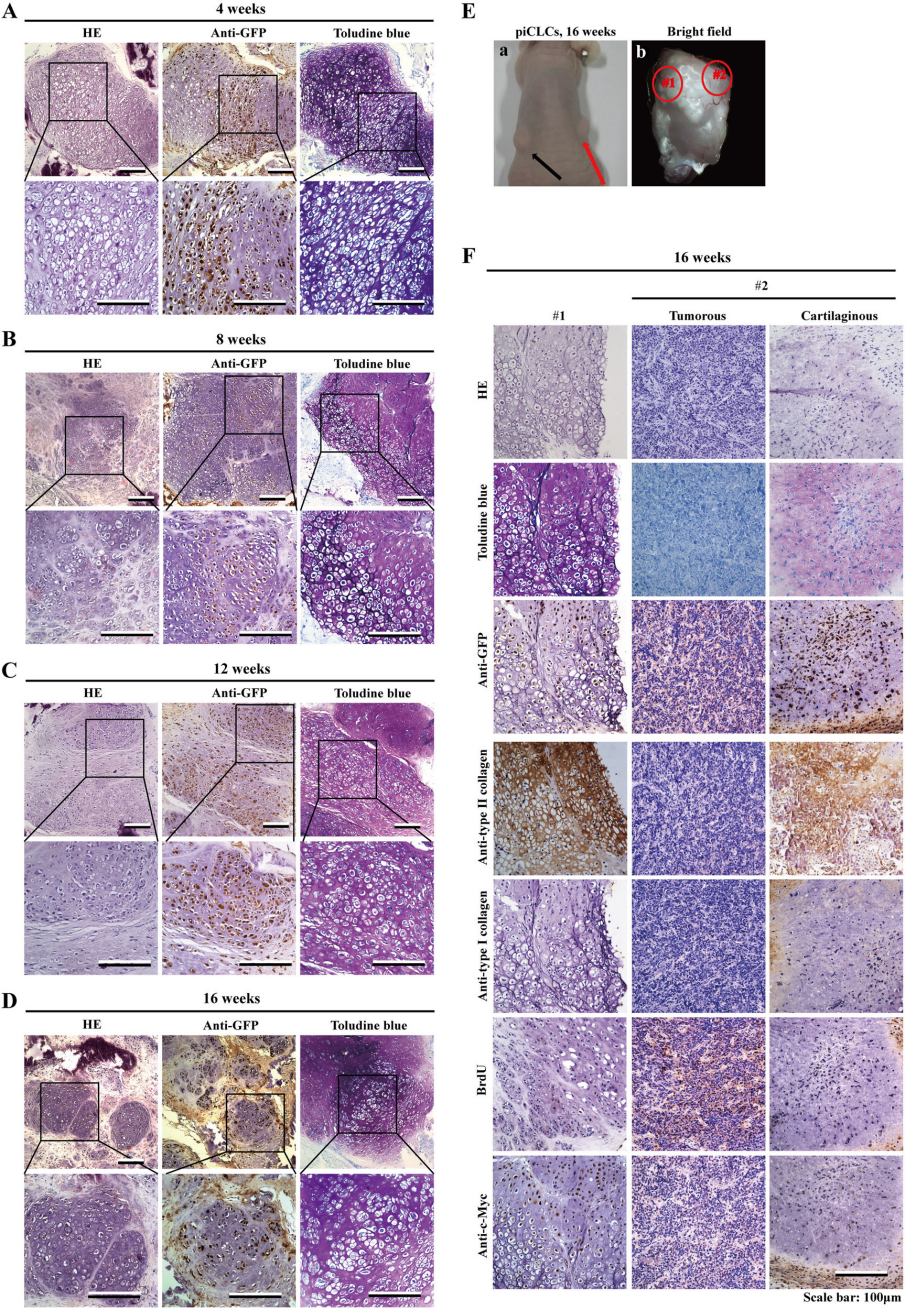
Prolonged of in vivo cartilage formation in nude mice. **a**–**d** Histology of grafted tissues 4 weeks (**a**), 8 weeks (**b**), 12 weeks (**c**), or 16 weeks (**d**) after injection of piCLCs (3 × 10^6^cells/site) into nude mice. Semiserial sections were stained with HE, toluidine blue and immunostained with anti-GFP antibody. For each tissue, lower-magnification images are shown above, and higher magnification images of boxed regions in the top panels are shown below. **e** Injection of piCLCs produced tumours (red arrow; **e**-**a**) at injected sites (16 weeks after injection). **e**-**a** Picture of nude mouse harbouring xenografts (arrows). **e**-**b** Picture of the xenograft (shown by red arrow in **e**-**a**) stripped from a nude mouse. Supplementary Fig. S6 and **f** show the histology of cartilage tissue and tumour tissue generated by piCLCs. **f** Semiserial sections of typical cartilage tissue (left column), tumorous portion (middle column) and cartilaginous portion (right column) formed from the transplanted piCLCs after injection were stained with HE and toluidine blue and immunostained with the indicated antibodies, as shown on the left. The typical cartilage tissue (left column) and the tumorous portion (middle column) and cartilaginous portion (right column) correspond to circled regions #1 and #2 in **e**-**b**, respectively

To further investigate whether piCLCs exhibit a chondrogenic phenotype in vivo as well as tumourigenicity in vivo during long-term cultivation in nude mice, we intradermally injected piCLCs (suspended in BD Matrigel) into the dorsal flank of nude mice (Table S1, Supplementary information). The xenografts became palpable at sites injected with piCLCs ~3 weeks after inoculation (data not shown), whereas the xenografts were never observed at sites injected with vector-expressing PEFs within 12 weeks (Table S1, Supplementary information). To examine the course of cartilage production in vivo, we collected tissues at 4, 8, 12 and 16 weeks after piCLC injection to perform histopathological analysis (Fig. 7). Our results revealed that 4, 8, 12 and 16 weeks after implantation into the dermal tissue of nude mice, the injected piCLCs generated substantial amounts of homogenous cartilage-like tissues, as indicated by HE staining and metachromatic staining with toluidine blue (Fig. 7a–d). We also observed lacuna formation, which is typical of cartilage (Fig. 7a–d and Fig. 7f, left column). The distribution of GFP-expressing cells almost exactly corresponded to regions with metachromatic staining, indicating that the injected GFP-positive piCLCs formed cartilage-like tissues (Fig. 7a, c, f). Positive immunostaining for GFP showed that the aforementioned homogenous cartilage-like tissues formed in the subcutaneous spaces of nude mice were derived from the injected GFP-positive piCLCs (Fig. 7a–d, f). Immunohistochemical analysis showed that the cartilage-like tissues generated by piCLCs strongly expressed type II collagen, but not type I collagen, suggesting that piCLC-derived cartilaginous tissues in vivo corresponded to hyaline cartilage rather than fibrocartilage (Fig. 7f, left column). Collectively, these findings demonstrate the cartilage-forming activities of piCLCs in vivo during long-term cultivation in nude mice and that hyaline cartilage–like tissues remain in vivo for at least 16 weeks.

Four, 8, 12 and 16 weeks after injection, we found solid nodules at 48 out of the 48 sites that were injected (100%) (Table S1, Supplementary information). Within 4 and 8 weeks after injection, histopathological analysis showed that piCLCs produced stable hyaline cartilage-like tissues without tumour formation at 25 injected sites (Fig. 1, Fig. 7a, b and Table S1, Supplementary information) for a sustained period of time in the subcutaneous spaces of nude mice. At 12 weeks after injection, we observed homogeneous cartilage formation at 16 injected sites, but we found tumour formation at 2 out of the 16 injected sites (Fig. 7c and Table S1, Supplementary information). At 16 weeks after injection, piCLC-injected sites continued to show homogeneous hyaline cartilage formation at 10 injected sites, whereas among 10 injected sites, 4 showed tumour formation (Fig. 7d, f and Table S1, Supplementary information).

We then followed the mice for tumour formation at 16 weeks after piCLC injection (Fig. 7e, f and Fig. S6 and Table S1, Supplementary information). Gross and histological examination revealed that xenografts (shown in Figs. 7e-b) consisted of both typical hyaline cartilage tissue (Fig. 7f, left column) and large tumorous portions, in which a very small portion of the atypical cartilaginous tissue was embedded (Fig. 7f, middle and right column and Fig. S6, Supplementary information). The red-circled region #1 in Fig. 7e-b of the stripped pig xenograft consisted largely of substantial amounts of typical and homogeneous hyaline cartilage-like tissues, as shown by metachromatic staining with toluidine blue, strongly positive expression of type II collagen, no type I collagen expression, and lacuna formation, which is typical of cartilage (Fig. 7f, left column). Although the sections of the small cartilaginous portion (shown in the circle marked with red-dotted line) embedded in a large tumorous portion (Fig. S6, Supplementary information) were positive on toluidine blue and type II collagen staining and negative for type I collagen (Fig. 7f, right column), the small cartilaginous portion did not display lacuna formation, suggesting that the small cartilaginous portion belonged to atypical cartilaginous tissues.

Low-power views revealed that that tumours consisted of a small portion of the untypical cartilaginous tissue embedded in a large tumorous portion (Fig. S6, Supplementary information and Fig. 7f, middle and right column). Under high-power observation, tumour cells had large nuclei with a coarse chromatin structure and one or two nucleoli, and the ratio of nucleus/cytoplasm was large (Fig. 7f, middle column). The typical hyaline cartilage-like tissues (Fig. 7f, left column) and the cartilaginous portion (Fig. S6, Supplementary information) that expressed type II collagen, but not type I collagen, did not display BrdU-positive cells in a representative field, indicating that piCLCs did not continued to replicate in cartilage at 16 weeks after injection (Fig. 7f, right and left column). However, the tumour cells, which had no positive expression of type I collagen and type II collagen, were highly proliferative, as indicated by the numerous BrdU-positive cells, (Fig. 7f, middle column). The same results obtained from that xenograft (Fig. 7e-b and Fig. S6, Supplementary information) were also observed in the other 5 xenografts with tumour formation (data not shown). The mixtures of cartilaginous tissues and tumorous tissues accounted for ~12% (6/51) of all xenografts (51).

## Discussion

The direct lineage conversion of somatic cells into various tissue-specific cell types (i.e., cardiomyocytes, neurons, hepatocytes and hematopoietic cells) and their stem-like precursors (i.e., neural stem cells, hepatic stem cells and blood progenitor cells) can be achieved without passing an intermediate pluripotent stage by introducing a set of defined transcription factors which are quite pivotal for the development of the destination cells^22^.

As cartilage has a limited capacity for repair when injured, the repair of articular cartilage defects needs alternative cell sources for a sufficient number of hyaline chondrocytes that can be transplanted to replace the defect tissues. A large number of autologous hyaline chondrocytes may be obtained by generating iPS cells, followed by redifferentiation into a chondrocytic lineage in the future^22–24^. However, the transplantation of redifferentiated chondrocytes is associated with a risk of teratoma formation due to the possible presence of residual undifferentiated cells^22–24^. The techniques of direct lineage reprogramming to chondrocytes have the great potential to resolve this problem by providing a sufficient number of hyaline chondrocytes to fill large defects^22–24^.

The direct lineage conversion strategy has also been successfully adopted in generating chondrocytes^10–12,25^. Mouse and human somatic cells (including fibroblasts) can be redirected into hyaline chondrocyte-like cells (i.e., iChon cells) without type I collagen gene expression by transducing a different set of transcription factors, including the combined transduction of two iPSC-reprogramming factors (c-Myc and Klf4) and one chondrogenic factor (SOX9)^10,11^, and a combination of only five genes (5 F pool)—c-Myc, BCL6, T, MITF and BAF60C^12^. In this study, we have generated pig hyaline chondrocyte-like cells (i.e., piCLCs) directly from PEF culture by transduction of one iPSC-reprogramming factor, c-MYC, which is a gene shared by the aforementioned two reprogramming systems for directly inducing chondrocytes from various somatic cells^10–12^. More importantly, mouse^10^, human^11^ and pig (this study) iChon cells produced histologically homogeneous hyaline cartilage-like tissues upon grafting in immunodeficient mice. Moreover, human iChon cells generated by the direct lineage reprogramming approach can survive and form cartilaginous tissue in the defects of articular cartilage of SCID rats^11^, suggesting that human iChon cells can be a candidate cell source for regenerative medicine to treat articular cartilage lesions caused by trauma, osteoarthritis and other diseases.

Next, we further analysed the potential reasons why piCLCs can be efficiently converted from PEFs by only c-Myc. The proto-oncogene c-Myc also plays important roles in the modulation of normal cell proliferation, differentiation, apoptosis, stem cell self-renewal, establishment and maintenance of pluripotency, and other processes^1–8^. Accumulated preliminary evidence shows that c-Myc might be essential for chondrocyte proliferation, differentiation, maturation and bone formation^15–21^. c-Myc is expressed in the proliferating and differentiating chondrocytes^15,18,19,21^. Enforced c-Myc expression promotes the proliferative expansion of chondrocytes^20^. Constitutive overexpression of c-Myc in immature chondrocytes maintains the cells in a proliferative state and blocks chondrocyte maturation^16,17,20^. Combined deletion of c-Myc and N-Myc in early limb bud mesenchyme gives rise to a severely hypoplastic limb skeleton that exhibits features characteristic of individual c-Myc and N-Myc mutants^20^. Therefore, c-Myc might play a critical role in the regulation of chondrocyte fates.

c-Myc is a critical reprogramming factor for iPS cells induced from animal and human somatic cells (including fibroblasts) by defined factors (Oct4, Sox2, c-Myc and Klf4)^13,14^. In addition, c-Myc is considered one of the crucial factors in the direct reprogramming of mouse fibroblasts into neural stem cells (NSCs)^26,27^ and chondrocytes when combined with Klf4 and SOX9^10,11^, while L-Myc, which can substitute for c-Myc, is indispensable for direct reprogramming of human fibroblasts into functional osteoblasts by defined factors (Oct4, L-Myc, Runx2 and Osterix)^28^. As mouse NSCs endogenously express high levels of Sox2, c-Myc and Klf4 as well as several intermediate reprogramming markers, Oct4 alone is sufficient to directly reprogram NSCs to iPS cells^29^. Our study showed that the in vitro–cultured pPr Ch and piCLCs expressed high levels of c-Myc (Fig. 3e) and chondrocyte marker genes (i.e., Col2a1, aggrecan, Sox5, Sox6 and Sox9) (Fig. 3c, e, f), whereas the parental PEFs of piCLCs did not express these genes (Fig. 3c, e, f) and other genes [i.e., Klf4 (data not shown) and SOX9(Fig. 3c)], suggesting that (1) PEFs do not endogenously express c-Myc, Klf4 and Sox9, which are pivotal for the development of the destination cells (i.e., iChon cells);^10,11^ and (2) c-Myc plays a crucial role in the control of pig chondrocyte fate because pPr Ch displayed high c-Myc expression (Fig. 3e). Thus, we rule out the possibility that piCLCs can be efficiently converted from PEFs by only c-Myc due to endogenous expression of Klf4 and SOX9. On the other hand, based on the aforementioned findings and information, we suspect that c-Myc serves as a critical transcription factor which is pivotal for the development and fate determination of the destination cells (i.e., piCLCs), which remains to be confirmed.

In summary, our findings reveal that (1) PEFs can be directly, rapidly and efficiently converted into piCLCs by ectopic expression of c-Myc alone; (2) at 45 out of the 51 injected sites, piCLCs produced stable homogeneous hyaline cartilage-like tissues without tumour formation and type I collagen expression after subcutaneous injection into nude mice, and piCLC-derived hyaline cartilage-like tissues remained in vivo for at least 16 weeks. Although significant challenges remain, the direct lineage reprogramming strategy may be a step toward the generation of person-, disease- and patient-specific chondrocytes without going through the process of generating iPS cells by a single gene. Additionally, please see“Supplementary Discussion”section for more discussion.

## Materials and methods

### Primary cell isolation and culture

Primary PEFs were prepared as previously described^9,30^. Briefly, a 35-day-old Tibetan miniature pig fetus (landrace) without head, limbs and internal organs was minced in phosphate buffered saline (PBS) and digested in Dulbeccos’ modified Eagle’s medium (DMEM) (Gibco) supplemented with 15% fetal bovine serum (FBS) (PAA), 1% penicillin-streptomycin (Gibco), 0.32 mg/ml collagenase IV (Sigma-Aldrich) and 2500 IU/ml DNase I (Gibco) for 6 h at 39 ℃. The cells were then centrifuged at 1000 rpm for 5 min, followed by suspension in DMEM supplemented with 15% FBS, 0.5% penicillin-streptomycin, 5% L-glutamine (Gibco) and 2.5% pyruvate (Gibco). PEFs and PDFs were cultured and maintained in DMEM medium supplemented with 10% FBS, 1 mM L-glutamine and 1% non-essential amino acids (Gibco) in a humidified incubator with 5% CO_2_ at 39 °C. PEFs at passage 1 were trypsinized and stored in liquid nitrogen until future use.

Primary chondrocytes of mice and pig were isolated as described previously^10,31^. The ventral sections of rib cages or the epiphyseal sections of humerus and femur were dissected from newborn mice or pig in PBS. Adherent soft tissues were removed from the cartilage after digestion with 2 mg/ml collagenase type II (Sigma-Aldrich) in DMEM at 37 °C for 30 min. Chondrocytes were released from the cartilage by soaking the tissue in fresh collagenase medium for 4-5 h. Released cells were collected by centrifugation (200 g for 10 min at 4 °C) and resuspended in the fresh medium. Cells were seeded into 60 mm or 100 mm dishes and cultured in DMEM supplemented with 10% FBS (passage 1).

### Plasmids, lentiviral production and transduction for stable cell lines

The cDNA of human c-Myc gene was inserted downstream of EF-1α promoter and upstream of IRES-EGFP of lentivirual vector to generate the lentiviral expression vector of pLenti-EF-1α-c-Myc-IRES-EGFP^32^, while the lentiviral packaging plasmids psPAX2 and pMD2.G were kindly provided by Dr. Didier Trono (University of Geneva, Geneva, Switzerland). To generate stable cell lines, recombinant lentiviruses [named as LV-EF-1α-IRES-EGFP (LV-con, used as vector control) and LV-EF-1α-c-Myc-IRES-EGFP (LV-c-Myc)] were generated as previously described^9^, and subsequently used to transduce PEFs to generate vector- or c-Myc-expressing PEFs, respectively. The infecting efficiency was estimated by EGFP assay and FACS analysis, while the successful overexpression of c-Myc was verified by immunofluorescence and Western blot assay.

### RNA isolation, reverse transcription and qRT-PCR

For mRNA analyses, total RNA from PEFs, piCLCs and primary chondrocytes were extracted using Trizol Reagent (TaKaRa) according to the protocol provided by the manufacturer. Total RNA was reversely transcribed with the PrimeScript RT reagent Kit (TaKaRa). qRT-PCR for the expression of mRNA analysis was performed using SYBR Green qRT-PCR master mix (TaKaRa) on a Stratagene Mx3005P qRT-PCR System (Agilent Technologies, Santa Clara, CA). GAPDH was used for normalization. The primers used for the amplification of the indicated genes were listed in Supplementary information, Table S2. All samples were normalized to internal controls and fold changes were calculated through relative quantification (2^−△△Ct^).

### Western blot analysis

Protein lysates extracted from PEFs, piCLCs and primary chondrocytes were separated by sodium dodecyl sulfate polyacrylamide gel electrophoresis, and transferred to a polyvinylidene difluoride membrane. The blots were probed with the indicated primary antibodies, followed by HRP (horseradish peroxidase)-labeled secondary antibodies. The hybridization signal was detected using enhanced chemiluminescence (Cat.No: KGP1122, KeyGEN BioTECH). β-actin was used as a loading control. The antibodies used in this study were shown in Supplementary information, Table S3.

### Immunofluorescent (IF) staining

IF staining was performed according to standard procedures and manufacturer recommendations. Briefly, cells grown on coverslips were rinsed with PBS and fixed with cold 4% paraformaldehyde for 5 min at room temperature. Subsequently, the cells were blocked with Triton X-100 at a concentration of 0.3% for 30 min and incubated with the indicated primary antibodies, and subsequently incubated with secondary goat anti-mouse or goat anti-rabbit (Invitrogen). Finally, the coverslips were counterstained with DAPI (Sigma-Aldrich) for 5 min to visualize the nuclei, and then imaged with a confocal laser-scanning microscope (Olympus FV1000). Data were processed with Adobe Photoshop 7.0 software. The antibodies used here were shown in Supplementary information, Table S3.

### CCK-8 assay and colony formation assay

Cell Counting Kit-8 (CCK-8) was used to evaluate the cell proliferation. 2000 cells were plated in 96-well plates and cultured in DMEM medium containing 10% FBS for 6 days. In general, 10 μl CCK-8 solution was added to each plate well, and then cells were incubated for 2 h in 37 °C. The cell viability was revealed by the absorbance which was measured at 450 nm. Colony formation assay was previously fully described^33,34^.

### Cell-cycle analysis and EdU incorporation assay

Cell-cycle analyse and EdU incorporation assay were performed according to a previous description^35^. For cell-cycle analysis, a total number of 5 × 10^6^ cells were harvested after 48 h incubation and then washed with cold PBS. The cells were further fixed with 70% ice-cold ethanol at 4 °C overnight. After incubation with PBS containing 10 mg/ml propidium iodide (Sigma-Aldrich) and 0.5 mg/ml RNase A (Roche) for 15 min at 37 °C, fixed cells were washed with cold PBS three times. Cell cycle analysis was carried out using the BDFACSDiva™. The population of cells in each of the G1, S, M and G2 phases was determined for at least 250,000 cells with doublet discrimination. Analysis of cell cycle position was performed using the BDFACSDiva software.

For EdU incorporation assay, the proliferating cells were examined using Click-iT™ EdU Imaging Kits (Invitrogen) according to the manufacturer’s protocol. Briefly, after incubation with 10 mM EdU for 2 h, cells were fixed with 4% paraformaldehyde, permeabilized in 0.3% Triton X-100 and stained with Alexa Fluor 594 azide. Hoechst 33342 was used to stain cell nuclei for 10 min. The number of EdU-positive cells was counted under a fluorescent microscope in five random fields. All assays were independently performed for three times.

### Soft agar colony formation assay

A cell suspension (2 × 10^3^ cells) in 2 ml DMEM supplemented with 10% FBS and 0.3% agar (Sigma-Aldrich) were layered onto 6-cm culture plates (Corning, NY, USA) containing 2 ml DMEM with 10% FBS and 0.6% agar. Plating was carried out in triplicate and repeated at least three times. After 21 days of growth, colonies were photographed and accounted.

### Determination of karyotypes

Karyotypes of piCLCs, PEFs and vector-expressing cells were determined with quinacrine-DAPI staining at the Technology Center of Prenatal Diagnosis and Genetic Diseases Diagnosis, Department of Obstetrics and Gynecology, Nanfang Hospital, Southern Medical University, Guangzhou, China.

### Xenograft experiments in nude mice

The animal experiments were carried out in strict accordance with the recommendations in the Guide for the Care and Use of Laboratory Animals of the Southern Medical University. The animal protocol was approved by the Committee on Ethics of Animal Experiments of the Southern Medical University. Nude mice were purchased from the Model Animal Research Center of Nanjing University, and housed in microisolator cages under aseptic conditions. PEFs harboring pLenti-EF-1α-c-Myc-IRES-EGFP or pLenti-EF-1α-IRES- EGFP transgene were resuspended in a mix of PBS and BD Matrigel (BD Biosciences) (1:1), and then subcutaneously injected into the dorsal flanks of 4-week old nude mice (3 × 10^6^cells/site). Mice were sacrificed at the indicated time point, and then the injected sites were dissected from the mice. The majority of xenograft tissue samples were fixed in 4% paraformaldehyde, dehydrated, embedded in paraffin, and sectioned. Other xenograft tissue samples were stored in −80 °C for future use. All surgery was performed under sodium pentobarbital anesthesia, and all efforts were made to minimize suffering of animals.

### Whole animal and xenograft fluorescence imaging

The protocols for whole-animal fluorescence imaging to noninvasively detect GFP expression by the Xenogen IVIS Lumina II Imaging System (Xenogen Corp., Alameda, CA, USA) were previously well described^33,36–39^. GFP expression in the isolated xenografts from nude mouse was assayed under stereo fluorescentmicroscope (Nikon, AZ100).

### Histological analysis and immunohistochemistry

For histology analysis, xenograft tissues were fixed with 4% paraformaldehyde in PBS, embedded in paraffin, cut into 5 μm thick sections, and then deparaffinized, followed by hematoxylin and eosin staining (H&E staining) according to standard procedures, as described previously^33,39^. The immunohistochemical staining procedure followed the standard streptavidin-peroxidase (SP) protocol, as described previously^33,39^ Antigen retrieval was achieved by high-pressure treatment in citrate buffer (pH 6.0) and boiling for 2 min. Endogenous peroxidase and non-specific staining were blocked by with H_2_O_2_ and 1% BSA for 15 min at room temperature, respectively. The sections were then incubated with the indicated primary antibodies (Supplementary information, Table S3) overnight at 4 °C and subsequently incubated with secondary antibodies. The complex was visualized with DAB and counterstained with hematoxylin. The antibodies used here were shown in Supplementary information, Table S3.

### Toluidine blue staining

Cells or tissue sections were fixed with 10% neutral buffered formalin at 25 °C for 10 min, washed with distilled water, incubated with 0.05% toluidine blue solution (Sigma) for 30 min at 25 °C, and washed 3 times with distilled water.

### Alcian blue staining

Cells or tissue sections were fixed with methanol at −20 °C for 2 min, incubated with 0.1% alcian blue (Sigma) in 0.1 N HCl for 2 h at 25 °C, and washed 3 times with distilled water.

### Statistical analysis

All data were presented as mean ± SD. Statistical analysis was performed using a SPSS 13.0 software package. Values are statistically significant at *P < 0.05; **P < 0.01.

## Supplementary information

Supplemental material
