# Supplementary material for: Direct conversion of pig fibroblasts to chondrocyte-like cells by c-Myc
Source: Cell Death Discov. 2019 Jan 18;5:55. doi: 10.1038/s41420-018-0136-4 (PMC6338791; doi:10.1038/s41420-018-0136-4)
Supplement: Supplementary file 1 — Supplemental material [file 41420_2018_136_MOESM1_ESM.docx]

**SUPPLEMENTARY INFORMATION**

**This supplementary files include:**

Supplementary Introduction

Supplementary Results

Supplementary Discussion

Supplementary references (for supplementary files)

Supplementary Figure S1–S6 (legends included)

Supplementary Tables S1–S3

**Supplementary Introduction**

c-Myc belongs to the Myc family of transcription factors, which also includes N-Myc and L-Myc. c-Myc is believed to regulate the expression of 15% of all genes. By modifying the expression of its target genes, c-Myc plays important roles in the control of normal cell proliferation, growth, differentiation, [apoptosis](http://en.wikipedia.org/wiki/Apoptosis), survival, [stem cell](http://en.wikipedia.org/wiki/Stem_cell) self-renewal, establishment and maintenance of pluripotency, and other processes^1-8^.

Broadly implicated in oncogenesis, the MYC family of proto-oncogenes are among the most studied genes in cancer^6^. c-Myc is a very strong proto-oncogene and is often upregulated in many types of cancers. In vitro studies of c-Myc have revealed its potential to malignantly transform normal embryonic fibroblasts and mammary epithelial cells^1,6,9-12^, while transgenic c-Myc overexpression in various tissues of transgenic mice is sufficient to drive oncogenesis (e.g., lymphomas, mammary carcinomas and liver cancer), illustrating the potency of the c-Myc oncogene^1,6,13-15^. Against this background, we choose the proto-oncogene c-Myc to induce in vitro the neoplastic transformation of pig fibroblasts, which lays a solid foundation for generating the transgenic pig models of human tumours.

Our previous study is first to reveal that the enforced expression of c-Myc in porcine embryonic fibroblasts (PEFs) triggered epithelial-like morphological conversion and mesenchymal-epithelial transition (MET) via F-actin reorganization and RhoA/Rock pathway inactivation^16^. In our pilot experiment, we u[nexpectedly](http://www.so.com/link?url=http%3A%2F%2Fdict.youdao.com%2Fsearch%3Fq%3Dunexpectedly%26keyfrom%3Dhao360&q=unexpectedly&ts=1508065824&t=aed45c1da5d16ee1bccf0c7fa2f9ec9) found that c-Myc-expressing PEFs generated cartilage-like tissues when subcutaneously injected into nude mice. Mouse and human somatic cells (including fibroblasts) can be directly converted into chondrocyte-like cells by a different set of transcription factors, including the combined transduction of two reprogramming factors (c-Myc and Klf4) and one chondrogenic factor (SOX9)^17,18^, and a combination of only five genes (5F pool)—c-Myc, BCL6, T (also called BRACHYURY), MITF and BAF60C (also called SMARCD3)^19^, indicating that the aforementioned two reprogramming systems for directly inducing chondrocytes from various somatic cells share a common gene, c-Myc. Moreover, c-Myc is a critical reprogramming factor for induced pluripotent stem cells (iPS cells or iPSCs) reprogrammed from animal and human somatic cells (including fibroblasts) by defined factors (Oct4, Sox2, c-Myc and Klf4)^20,21^. Furthermore, increasing evidence demonstrates that the proto-oncogene c-Myc is also involved in chondrocyte proliferation, differentiation and maturation, as well as bone formation (see Discussion for details)^22-28^. These above-mentioned findings prompted us to suspect that PEFs can be directly converted into pig induced chondrocyte-like cells (piCLCs) by only c-Myc, which has never been reported.

In the present study, we examined whether piCLCs could be directly induced from PEFs pig fibroblasts by the re-expression of c-Myc alone. We also assessed the capability of the resulting directly converted piCLCs to contribute to cartilage-like tissue formation in vivo. We successfully induced piCLCs directly from PEF culture by the transduction of c-Myc alone. piCLCs produced cartilage-like tissue after subcutaneous injection into nude mice. Pig cartilage-like tissues remained for at least 16 weeks.

**Supplementary Results**

**1. Ectopic expression of c-Myc in PEFs improves cell proliferation capacity**

First, the primary PEFs (Fig. S1, Supplementary information) were made as fully described in the Materials and Methods. Second, f[or the above-mentioned reasons](http://www.englishforums.com/English/ForAboveMentionedReasons/njkqk/post.htm), both the c-Myc transgene and the EGFP gene were introduced into PEFs via lentivirus-mediated *in vitro* gene transfer to generate a stable PEF line expressing both EGFP and c-Myc transgenes (Fig. S2, Supplementary information). Nearly 100% infection efficiency was determined by EGFP assay (Fig. S2A, Supplementary information), while the successful enforced c-Myc expression in PEFs was determined by immunofluorescence staining (Fig. S2B, Supplementary information) and Western blot (Fig. S2C, Supplementary information), indicating that the stable PEF line expressing c-Myc transgene was successfully attained.

Our previous study revealed that the enforced expression of c-Myc in PEFs triggered epithelial-like morphological conversion and MET via F-actin reorganization and RhoA/Rock pathway inactivation^16^. To further determine the effects of c-Myc on PEF growth, a CCK8 assay, EdU assay, colony formation assay and cell cycle analysis by flow cytometry were performed to evaluate the proliferation ability of c-Myc-expressing PEFs. As shown in Fig. 1A,B, the results of CCK8 assay and EdU incorporation assay showed that c-Myc-expressing PEFs exhibited the significantly enhanced proliferation ability compared to vector-expressing PEFs. The colony formation assay showed that colony formation was observed in the LV-c-Myc group, but not the control group (Fig. 1C). Following observation of c-Myc–mediated growth enhancement, cell cycle analysis by flow cytometry was used to analyse cell-cycle distribution of c-Myc-expressing PEFs. Compared with LV-con, PEFs expressing c-Myc displayed a significantly decreased percentage of cells in G1 phase and more cells in S phase (Fig. 1D), suggesting that c-Myc overexpression induced G1/S transition in PEFs. Thus, c-Myc-expressing PEFs, which underwent an epithelial-like morphological change and MET induced by the enforced c-Myc expression^16^, displayed enhanced proliferation ability in vitro compared with vector-expressing PEFs.

**2. The establishment of the methods for isolating, culturing and identifying the chondrocytes of mice and pigs**

First, the primary chondrocytes of mice and pigs (Fig. S1, Supplementary information) were made as described in the Materials and Methods. Secondly, prior to assessing the chondrocyte characteristics of c-Myc-expressing PEFs, we successfully set up the identification system for mouse and pig chondrocytes based on cell morphology, toluidine blue staining, alcian blue staining, and immunofluoresence staining (Figs S1 and S4, Supplementary information)^16^. As shown in Supplementary information, Fig. S1A, the cultured mouse primary chondrocytes (mPr Ch) made in this study displayed a polygonal morphology, which is typical of cultured chondrocytes, as further confirmed by toluidine blue and alcian blue staining (Fig. S4A, Supplementary information), and immunocytochemistry assay (Fig.S4C, Supplementary information). In this study, pig primary chondrocytes (pPr Ch) were prepared from newborn pig rib (Fig. S1B, Supplementary information), but unlike mPr Ch with the aforementioned typical shape, we found that these cultured cells (obtained from pig rib) that exhibited a triangle- or short spindle-shaped morphology (Fig. S1B, Supplementary information) were positively stained by toluidine blue (Fig. S4B, Supplementary information) and alcian blue (Fig. S4B, Supplementary information) and stained positive for type II collagen (Fig. S4C, Supplementary information) and aggrecan (Fig. S4C, Supplementary information), but were negative for type I collagen (Fig. S4C, Supplementary information), suggesting that these cultured triangle- or short spindle-shaped cells isolated from pig rib displayed chondrocyte characteristics. Moreover, newborn porcine cartilage tissues were positively stained by toluidine blue and alcian blue (Fig. S4D, Supplementary information). Although the cultured pPr Ch showed the above-mentioned atypical morphology of chondrocytes, pPr Ch contained in newborn porcine cartilage tissues displayed the polygonal or round morphology, which is typical of cultured chondrocytes (Fig. S4D, Supplementary information). Thus, the methods for isolating, culturing and identifying the chondrocytes of pigs were successfully established.

**3. Chondrocyte marker gene expression analyses of piCLCs-S**

As shown in Fig. 3 and Fig. 4, almost 100% of mixed piCLCs with the chondrogenic phenotype displayed metachromatic toluidine blue and alcian blue staining with different staining intensities, suggesting the heterogeneity of mixed piCLC populations. To investigate whether single clones derived from mixed piCLCs (hereinafter referred to as piCLCs-S) exhibited a chondrogenic phenotype in vitro, the limiting dilution technique was employed to isolate single clones. We found that the single clones composed of homogeneously short spindle-shaped cells or polygonal cells were almost all intensely and homogenously stained with toluidine blue and alcian blue (Fig. 5A,B). qRT-PCR showed that piCLCs-S specifically expressed chondrocyte-specific marker genes (i.e., Col2a1, aggrecan, Sox5, Sox6 and Sox9) (Fig. 5C). On the other hand, qRT-PCR analysis illustrated that piCLCs-S hardly expressed fibroblast-associated Col1a1 and Col1a2, which the parental vector-expressing PEFs expressed abundantly (Fig. 5D). Additionally, immunofluorescence staining revealed that piCLCs-S expressed type II collagen and aggrecan, but not type I collagen (Fig. 5E). Together, these findings illustrate that piCLCs-S with a chondrogenic phenotype in vitro exhibits homogeneity in cell m[orphology and staining intensity](https://www.researchgate.net/profile/Marcus_Kaiser2/publication/225054176_Morphological_Homogeneity_of_Neurons_Searching_for_Outlier_Neuronal_Cells/links/0fcfd502a97e95669d000000) compared with mixed piCLCs.

**4. Chondrocyte phenotypic stability assay, tumourigenicity assay and karyotype analysis for piCLCs**.

To determine whether piCLCs can maintain the chondrocyte phenotype during long-term culture, piCLCs were passaged every 2-4 days for up to 21 passages, followed by cell morphology assay, toluidine blue staining and alcian blue staining. As indicated in Fig. 6, there were no significant changes in cell morphology (Fig. 6A) and staining intensity (Fig. 6B) between passage 5 (P5) and passage 21 (P21) piCLCs, suggesting that piCLCs can maintain the chondrocytic phenotype during long-term subculture. Furthermore, Western blot analysis showed that the different generations of piCLCs constantly expressed type II collagen and aggrecan (Fig. 3E).

As shown in Fig.1, c-Myc-expressing PEFs exhibited significantly increased proliferation ability in vitro compared with vector-expressing PEFs and pPr Ch. Anchorage-independent colony formation is a hallmark of transformation and an in vitro correlate of tumourigenicity in vivo. A soft-agar colony formation assay was employed to evaluate the tumourigenicity of piCLCs in vitro. Human nasopharyngeal carcinoma CNE2 cells, as a positive control, displayed colony formation in soft agar (Fig. 6C), whereas piCLCs at the three indicated doses never formed colonies in soft agar (Fig. 6D), indicating that piCLCs have no capacity for anchorage-independent growth in vitro.

A karyotype analysis showed that P5 piCLCs (Fig. 6E-c) and P21 piCLCs (Fig. 6E-d) had normal karyotypes, indicating that piCLCs can maintain normal karyotypes during long-term subculture. Additionally, the parent PEFs (Fig. 6E-a) and vector-expressing PEFs (Fig. 6E-b) had normal karyotypes.

**Supplementary Discussion**

In this study, we have efficiently accomplished direct conversion of PEFs into piCLCs in vitro by the ectopic expression of c-Myc alone. Although the mixtures of cartilage tissues and tumorous tissues accounted for ~12% (6/51) of all xenografts (51), piCLCs survived and produced stable homogenous hyaline cartilage-like tissues without tumour formation at 45 out of the 51 injected sites after subcutaneous injection into nude mice, and hyaline cartilage–like tissues remained for at least 16 weeks.

The direct lineage conversion or direct lineage reprogramming of somatic cells into various tissue-specific cell types (i.e., cardiomyocytes, neurons, hepatocytes and hematopoietic cells) and their stem-like precursors (i.e., neural stem cells, hepatic stem cells and blood progenitor cells) can be achieved without passing an intermediate pluripotent stage by introducing a set of defined transcription factors which are quite pivotal for the development of the destination cells^29^. Although the conversion efficiencies are generally low at present, with only 0.005–30% of fibroblasts successfully converted into the destination cells, this technology may become quite useful for cell-based therapies against a variety of human diseases^29^.

Cartilage is a stiff yet flexible connective tissue found in many areas in the bodies of humans and other animals. It is composed of chondrocytes which produce a large amount of collagen fibre, an abundant basal substance rich in proteoglycans and elastin fibres. As cartilage has a limited capacity for repair when injured, the repair of articular cartilage defects needs alternative cell sources for a sufficient number of hyaline chondrocytes that can be transplanted to replace the defect tissues. A large number of autologous hyaline chondrocytes may be obtained by generating iPS cells, followed by redifferentiation into a chondrocytic lineage in the future^30-32^. However, the transplantation of redifferentiated chondrocytes is associated with a risk of teratoma formation due to the possible presence of residual undifferentiated cells^30-32^. The techniques of direct lineage reprogramming to chondrocytes have the great potential to resolve this problem by providing a sufficient number of hyaline chondrocytes to fill large defects^30-32^.

The direct lineage conversion strategy has also been successfully adopted in generating chondrocytes without passing through a stem cell state by the ectopic expression of defined lineage-specific transcription factors based on their master-regulatory roles in chondrogenesis^17-19,33^. Mouse and human somatic cells (including fibroblasts) can be redirected into hyaline chondrocyte-like cells (i.e., iChon cells) without type I collagen gene expression by transducing a different set of transcription factors, including the combined transduction of two iPSC-reprogramming factors (c-Myc and Klf4) and one chondrogenic factor (SOX9)^17,18^, and a combination of only five genes (5F pool)—c-Myc, BCL6, T (also called BRACHYURY), MITF and BAF60C (also called SMARCD3)^19^. In this study, we have successfully and efficiently generated pig hyaline chondrocyte-like cells (i.e., piCLCs) directly from PEF culture by transduction of one iPSC-reprogramming factor, c-MYC, which is a gene shared by the aforementioned two reprogramming systems for directly inducing chondrocytes from various somatic cells^17-19^. More importantly, mouse^17^, human^18^ and pig (this study) iChon cells produced histologically homogeneous hyaline cartilage-like tissues upon grafting in immunodeficient mice. Moreover, human iChon cells generated by the direct lineage reprogramming approach can survive and form cartilaginous tissue in the defects of articular cartilage of SCID rats^18^, suggesting that human iChon cells can be a candidate cell source for regenerative medicine to treat articular cartilage lesions caused by trauma, osteoarthritis and other diseases.

To dissect the minimum essential combination of defined factors, the effects of the different combinations of 4 reprogramming factors (Oct3/4, Sox2, c-Myc and Klf4) and SOX9 on direct chondrocyte-like conversion from fibroblasts have been fully evaluated^17^. The retroviral transduction of different combinations of a few defined factors, such as c-Myc, Klf4, Oct3/4 and Sox2 plus SOX9; c-Myc, Klf4 and Oct3/4 plus SOX9; c-Myc, Klf4 and Sox2 plus SOX9; and c-Myc and Klf-4 plus SOX9, can induce polygonal chondrogenic cells stained positively with toluidine blue directly from mouse dermal fibroblasts (MDFs) and mouse embryonic fibroblasts (MEFs)^17^, whereas retroviral transduction of defined factors, such as Klf4, Oct3/4 and Sox2 plus SOX9; c-Myc, Oct3/4 and Sox2 plus SOX9; c-Myc plus SOX9; Klf4 plus SOX9; and c-Myc plus Klf4, did not^18^. These results suggest that (1) in this reprogramming system, c-Myc and Klf4 are critical for the transformation of cells in MDF and MEF populations into chondrocyte-like cells with metachromatic toluidine blue staining and polygonal chondrocyte-like morphology, and (2) the minimum essential combination of c-Myc and Klf4 plus SOX9 can efficiently convert MDFs and MEFs into substantial numbers of chondrocyte-like cells with metachromatic toluidine blue staining and polygonal chondrocyte-like morphology. Moreover, human iChon cells were efficiently reprogrammed directly from human dermal fibroblasts (HDFs) by the same combination of three factors (c-Myc, Klf4 and SOX9)^18^. c-Myc and Klf4 are responsible for erasing the characteristics of fibroblasts during iPS cell induction by c-Myc, Klf4, Oct3/4 and Sox2^34^. The expression of fibroblast markers decreases first, followed by an increase in the expression of chondrocyte markers during the induction of mouse chondrogenic cells from MDFs by c-Myc, Klf4 and SOX9^17^. These findings suggest that c-Myc and Klf4 are involved in epigenetic events in MDFs and HDFs and enable SOX9 to direct cells to the chondrogenic lineage during the induction of iChon cells. Unexpectedly, we found that piCLCs were directly, rapidly and efficiently converted in vitro from PEFs by ectopic expression of c-Myc alone. Although all PEFs (from pig embryos), MDFs (from mouse skin), MEFs (from mouse embryo) and HDFs (from human skin) are fibroblasts, the data from this study and other labs clearly show that the ectopic expression of only c-Myc can efficiently convert iChon cells from PEFs, but not MDFs, MEFs or HDFs. We suspect that the above-mentioned differences may be partly caused by species specificity or cell type, which remains to be elucidated.

c-Myc is a critical reprogramming factor for iPS cells induced from animal and human somatic cells (including fibroblasts) by defined factors (Oct4, Sox2, c-Myc and Klf4)^20,21^. In addition, c-Myc is considered one of the crucial factors in the direct reprogramming of mouse fibroblasts into neural stem cells (NSCs)^35,36^ and chondrocytes when combined with Klf4 and SOX9^17,18^, while L-Myc, which can substitute for c-Myc, is indispensable for direct reprogramming of human fibroblasts into functional osteoblasts by defined factors (Oct4, L-Myc, Runx2 and Osterix) ^37^. As mouse NSCs endogenously express high levels of Sox2, c-Myc and Klf4 as well as several intermediate reprogramming markers, Oct4 alone is sufficient to directly reprogram NSCs to iPS cells^38^. Our study showed that the in vitro–cultured pPr Ch and piCLCs expressed high levels of c-Myc (Fig.3E) and chondrocyte marker genes (i.e., Col2a1, aggrecan, Sox5, Sox6 and Sox9) (Fig.3C,E,F), whereas the parental PEFs of piCLCs did not express these genes (Fig.3C,E,F) and other genes [i.e., Klf4 (data not shown) and SOX9(Fig.3C)], suggesting that (1) PEFs do not endogenously express c-Myc, Klf4 and Sox9, which are pivotal for the development of the destination cells (i.e., iChon cells)^17,18^; and (2) c-Myc plays a crucial role in the control of pig chondrocyte fate because pPr Ch displayed high c-Myc expression (Fig.3E). Thus, we rule out the possibility that piCLCs can be efficiently converted from PEFs by only c-Myc due to endogenous expression of Klf4 and SOX9. On the other hand, based on the aforementioned findings and information, we suspect that c-Myc serves as a critical transcription factor which is pivotal for the development and fate determination of the destination cells (i.e., piCLCs), which remains to be confirmed. Altogether, the aforementioned findings demonstrate that we still know very little about the functions of c-Myc in the control of chondrocyte fate and its underlying mechanisms, which prompts us to fully dissect the roles of c-Myc in the fate regulation of chondrocytes and the underlying mechanisms that may contribute to why piCLCs can be reprogrammed directly from PEFs by c-Myc alone.

One goal in cartilage tissue engineering, drug testing and gene therapy is to obtain expandable chondrogenic cells that do not lose their chondrogenic phenotypes, but cultured chondrocytes do not rapidly proliferate in vitro for a sustained period of time and reach senescence rapidly to lose proliferation ability after passaging^30-32^. Chondrogenic progenitor cells collected from human osteoarthritic cartilage reach senescence after expansion^39^. Growth curve analysis has shown that mouse iChon cells grow exponentially for more than 45 days, while the parental MDFs and primary chondrocytes stop growing at 15 and 21 days, respectively^17^. In this study, piCLCs displayed enhanced proliferation ability in vitro compared with the parental PEFs and pPr Ch (Fig.1), while during long-term culture and passaging, the ability to rapidly proliferate in vitro was maintained in piCLCs that did not lose their chondrogenic properties (Fig.6A，B). Thus, iChon cells may provide a potential source of expandable chondrogenic cells for drug testing, gene therapy and cell-based therapy.

MET, accompanied by epithelial-like morphological changes, is a critical initiating event during the derivation of iPS cells from fibroblasts^40-43^. Our previous study was the first to reveal that the enforced expression of c-Myc in PEFs triggered epithelial-like morphological conversion and MET via F-actin reorganization and RhoA/Rock pathway inactivation^16^. In this study, 10 days after infection, c-Myc-expressing PEFs (i.e., piCLCs) displayed typical short spindle-like or polygonal-like morphology (Fig.4A,B) with intense toluidine blue and alcian blue staining compared with vector-expressing PEFs (Supplementary information, Fig.S5), which had a fibroblast-like appearance, while mouse^17^ and human^18^ iChon cells which were converted from fibroblasts by transduction of c-Myc, Klf4 and SOX9 also exhibit typical polygonal chondrocyte-like morphology, suggesting that iChon cells^17,18^ and piCLCs (this study) with chondrogenic properties have undergone epithelial-like morphological conversion. Altogether, these findings suggest that MET may be a critical initiating event during the derivation of iChon cells from mouse and human fibroblasts, and piCLCs from PEFs, which remains to be fully investigated.

Although c-Myc plays important roles in many normal physiological processes^1-8^, c-Myc is a very strong proto-oncogene and is often upregulated in many types of cancers^6^. Cell type conversion through iPS cells is associated with two different risks of tumour formation: one is the risk of teratoma formation associated with the pluripotency of iPS cells and the other is associated with the transduction of the reprogramming factors. piCLCs (this study) and iChon cells^17,18^ are associated with the latter risk because c-Myc (for piCLCs) or c-Myc and Klf4 (for iChon cells) are used. Although piCLCs produced stable homogenous hyaline cartilage-like tissues without tumour formation at 45 out of the 51 injected sites for a sustained period of time (i.e., 4, 8, 12 and 16 weeks after injection) in the subcutaneous spaces of nude mice and piCLCs didn’t display the capacity for the anchorage-independent growth in vitro, the mixtures of cartilage tissues and tumorous tissues accounted for ~12% (6/51) of all xenografts (51). Moreover, a few iChon cell lines formed mixtures of cartilaginous tissues and tumorous tissues, while other iChon cell lines have produced stable hyaline cartilage–like tissues without tumour formation for a sustained period of time in the subcutaneous spaces of nude mice^17^. However, as long as the c-Myc transgene is present in piCLCs, it is possible that these cells will eventually become tumorigenic. To decrease the risk of tumourigenicity, the c-Myc construct should be transiently expressed to induce piCLCs and then eliminated after a sufficient number of piCLCs are generated. Safer iPS cells have recently been generated by using integration-free vectors, such as episomal plasmid vectors^44^ and Sendi virus vectors^45^ . Therefore, it will be ideal to produce piCLCs by transient expression of c-Myc using integration-free vectors to minimize the risk of tumour formation. On the other hand, L-Myc, another Myc family member, can substitute for c-Myc to efficiently induce safer iPS cells^46^ and safer functional osteoblasts^37^ from fibroblasts because L-Myc displays lower tumourigenicity compared with WT c-Myc. These data encourage us to fully explore whether safer piCLCs can be converted from PEFs by the enforced expression of L-Myc in place of c-Myc in the near future.

In summary, our findings reveal that (1) PEFs can be directly, rapidly and efficiently converted into piCLCs by ectopic expression of c-Myc alone; (2) at 45 out of the 51 injected sites, piCLCs produced stable homogeneous hyaline cartilage-like tissues without tumour formation and type I collagen expression after subcutaneous injection into nude mice, and piCLC-derived hyaline cartilage-like tissues remained in vivo for at least 16 weeks; and (3) mixtures of cartilaginous tissues and tumorous tissues were observed at 6 out of the 51 injected sites. The following issues still need to be fully investigated: (1) the detailed molecular mechanisms involved in the direct conversion of piCLCs from PEFs by c-Myc alone; (2) whether other types of somatic cell obtained from pig, mouse or human can be directly reprogrammed into expandable chondrocytes with chondrogenic potential by c-Myc alone; and (3) whether L-Myc can substitute for c-Myc to derive safer piCLCs from PEFs. Although significant challenges remain, the direct lineage reprogramming strategy may be a step toward the generation of person-, disease- and patient-specific chondrocytes without going through the process of generating iPS cells by a single gene.

**Supplementary references**

1. Dang, CV. MYC on the path to cancer. *Cell* . **149**,22-35(2012).

2. Gustafson, WC.et al. Myc proteins as therapeutic targets. *Oncogene* . **29**,1249-1259(2010).

3. Hynes, NE. et al. Key signalling nodes in mammary gland development and cancer: Myc. *Breast cancer research : BCR*.**11**,210(2009).

4. Larsson, LG. et al. The Yin and Yang functions of the Myc oncoprotein in cancer development and as targets for therapy. *Experimental cell research* . **316**,1429-1437(2010).

5.Laurenti, E. et al. Myc's other life: stem cells and beyond. *Current opinion in cell biology* . **21**,844-854(2009).

6. Meyer, N. et al. Reflecting on 25 years with MYC. *Nature reviews Cancer* . **8**,976-990(2008).

7. Smith, K. et al. Myc transcription factors: key regulators behind establishment and maintenance of pluripotency. *Regenerative medicine* . **5**,947-959(2010).

8. Soucek, L. et al. The ups and downs of Myc biology. *Current opinion in genetics & development* . **20**,91-95(2010).

9.Cowling, VH. et al. E-cadherin repression contributes to c-Myc-induced epithelial cell transformation. *Oncogene* . **26**.3582-3586(2007).

10.Land, H. et al.Tumorigenic conversion of primary embryo fibroblasts requires at least two cooperatingoncogenes. *Nature* . **304**,596-602(1983).

11. Liu, M. et al. Casimiro MC, Wang C *et al.* p21CIP1 attenuates Ras- and c-Myc-dependent breast tumor epithelial mesenchymal transition and cancer stem cell-like gene expression in vivo. *Proceedings of the National Academy of Sciences of the United States of America* . **106**,19035-19039(2009).

12. Trimboli, AJ. et al*.* Direct evidence for epithelial-mesenchymal transitions in breast cancer. *Cancer research* . **68**,937-945(2008).

13. Adams, JM. et al. The c-myc oncogene driven by immunoglobulin enhancers induces lymphoid malignancy in transgenic mice. *Nature* . **318**,533-538(1985).

14. Chesi, M. et al. Robbiani DF, Sebag M *et al.* AID-dependent activation of a MYC transgene induces multiple myeloma in a conditional mouse model of post-germinal center malignancies. *Cancer cell* . **13**,167-180(2008).

15. Leder, A. et al. Pattengale PK, Kuo A, Stewart TA, Leder P. Consequences of widespread deregulation of the c-myc gene in transgenic mice: multiple neoplasms and normal development. *Cell* 1986. **45**,485-495(1986).

16. Shi, JW. et al. The enforced expression of c-Myc in pig fibroblasts triggers mesenchymal-epithelial transition (MET) via F-actin reorganization and RhoA/Rock pathway inactivation. *Cell cycle (Georgetown, Tex)* . **12**,1119-1127(2013).

17. Hiramatsu, K. et al. Sasagawa S, Outani H, Nakagawa K, Yoshikawa H, Tsumaki N. Generation of hyaline cartilaginous tissue from mouse adult dermal fibroblast culture by defined factors. *The Journal of clinical investigation* . **121**,640-657(2011).

18. Outani, H. et al. Okada M, Yamashita A, Nakagawa K, Yoshikawa H, Tsumaki N. Direct induction of chondrogenic cells from human dermal fibroblast culture by defined factors. *PloS one* . **8**,e77365(2013).

19. Ishii, R. et al. Placenta to cartilage: direct conversion of human placenta to chondrocytes with transformation by defined factors. *Molecular biology of the cell* . **23**,3511-3521(2012).

20. Takahashi, K. et al. Induction of pluripotent stem cells from mouse embryonic and adult fibroblast cultures by defined factors. *Cell* . **126**,663-676(2006).

21. Takahashi, K. et al. Induction of pluripotent stem cells from adult human fibroblasts by defined factors. *Cell* . **131**,861-872(2007).

22. Farquharson, C. et al. The proto-oncogene c-myc is involved in cell differentiation as well as cell proliferation: studies on growth plate chondrocytes in situ. *Journal of cellular physiology*.**152**,135-144(1992).

23. Iwamoto, M. et al*.* Expression and role of c-myc in chondrocytes undergoing endochondral ossification. *The Journal of biological chemistry* . **268**,9645-9652(1993).

24. Piedra, ME. et al. c-Myc overexpression increases cell size and impairs cartilage differentiation during chick limb development. *Cell growth & differentiation : the molecular biology journal of the American Association for Cancer Research* . **13**,185-193(2002).

25. Tsuji, Y. et al. Cell proliferation and differentiation of cultured chondrocytes isolated from growth plate cartilage of rat rib. *Nihon geka hokan Archiv fur japanische Chirurgie* . **64**,50-63(1995).

26. Wang,Y. et al. Expression and subcellular localization of the Myc superfamily proteins: c-Myc, Max, Mad1 and Mxi1 in the epiphyseal plate cartilage chondrocytes of growing rats. *Cellular and molecular biology (Noisy-le-Grand, France)* . **43**,175-188(1997).

27. Zhou, ZQ. et al. Sequential and coordinated actions of c-Myc and N-Myc control appendicular skeletal development. *PloS one*. **6**,e18795(2011).

28. Alonge, TO. et al. Oncogene expression in the peri-articular osteophytes. *West African journal of medicine*.**23**,187-190(2004).

29. Morris, SA. Direct lineage reprogramming via pioneer factors; a detour through developmental gene regulatory networks. *Development (Cambridge, England)* . **143**,2696-2705(2016).

30. Driessen, BJH. et al. Cellular reprogramming for clinical cartilage repair. *Cell biology and toxicology* . **33**,329-349(2017).

31. Jayasuriya, CT. et al. The influence of tissue microenvironment on stem cell-based cartilage repair. *Annals of the New York Academy of Sciences* .**1383**,21-33(2016).

32. Tsumaki, N. et al. iPS cell technologies and cartilage regeneration. *Bone* 2015. **70**,48-54(2015).

33. Wang, Y. et al*.* Reprogramming of Dermal Fibroblasts into Osteo-Chondrogenic Cells with Elevated Osteogenic Potency by Defined Transcription Factors. *Stem cell reports* . **8**,1587-1599(2017).

34. Wernig, M. et al. c-Myc is dispensable for direct reprogramming of mouse fibroblasts. *Cell stem cell* . **2**,10-12(2008).

35. Han, DW. et al. Direct reprogramming of fibroblasts into neural stem cells by defined factors. *Cell stem cell* . **10**,465-472(2012).

36. Their, M. et al*.* Direct conversion of fibroblasts into stably expandable neural stem cells. *Cell stem cell* . **10**,473-479(2012).

37. Yamamoto, K*.* et al. Direct conversion of human fibroblasts into functional osteoblasts by defined factors. *Proceedings of the National Academy of Sciences of the United States of America*. **112**,6152-6157(2015).

38. Kim, JB. et al. Sebastiano V, Wu G *et al.* Oct4-induced pluripotency in adult neural stem cells. *Cell* . **136**,411-419(2009).

39. Koelling ,S. et al*.* Migratory chondrogenic progenitor cells from repair tissue during the later stages of human osteoarthritis. *Cell stem cell* . **4**,324-335(2009).

40. Li,R. et al*.* A mesenchymal-to-epithelial transition initiates and is required for the nuclear reprogramming of mouse fibroblasts. *Cell stem cell* . **7**,51-63(2010).

41. Liu, X. et al*.* Sequential introduction of reprogramming factors reveals a time-sensitive requirement for individual factors and a sequential EMT-MET mechanism for optimal reprogramming. *Nature cell biology* . **15**,829-838(2013).

42. Polo, JM. et al. When fibroblasts MET iPSCs. *Cell stem cell* . **7**,5-6(2010).

43. Samavarchi-Tehrani, P. et al. Functional genomics reveals a BMP-driven mesenchymal-to-epithelial transition in the initiation of somatic cell reprogramming. *Cell stem cell* .**7**,64-77(2010).

44. Okita, K. et al*.* A more efficient method to generate integration-free human iPS cells. *Nature methods* . **8**,409-412(2011).

45. Seki, T. et al. Generation of induced pluripotent stem cells from human terminally differentiated circulating T cells. *Cell stem cell* . **7**,11-14(2010).

46. Nakagawa, M. et al. Promotion of direct reprogramming by transformation-deficient Myc. *Proceedings of the National Academy of Sciences of the United States of America* . **107**,14152-14157(2010).


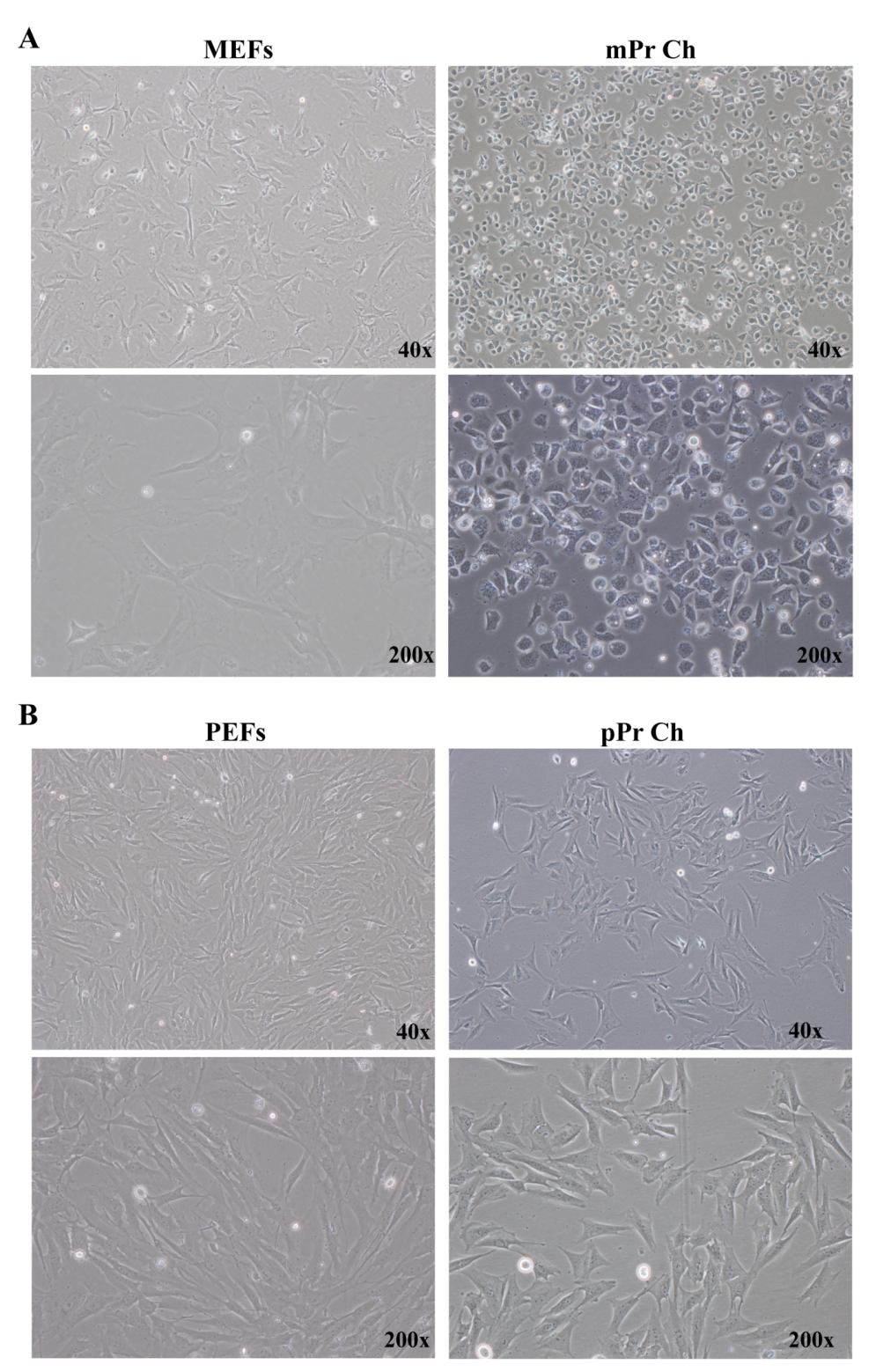
**Supplementary Figures**

**Fig. S1** [**Isolation and culture of primary**](http://www.so.com/link?m=awd3M1UA15Ngd0cgo1MBXQlwt8o0G1pKRHorjJR%2BqLQKhXCnpOR4KmS0%2BLDBxYmc87W5e51l2cumEidPg0m6om8kY0Hp1eLFv%2BzhIh0%2FO2I%2BfjuoSBwaDqA%3D%3D)**embryonic fibroblasts and primary chondrocytes.**

(**A**) Pictures of primary mouse embryonic fibroblasts (MEFs) and primary mouse chondrocytes (mPr Ch).

(**B**) Pictures of primary porcine embryonic fibroblasts (PEFs) and primary porcine chondrocytes (pPr Ch).

**
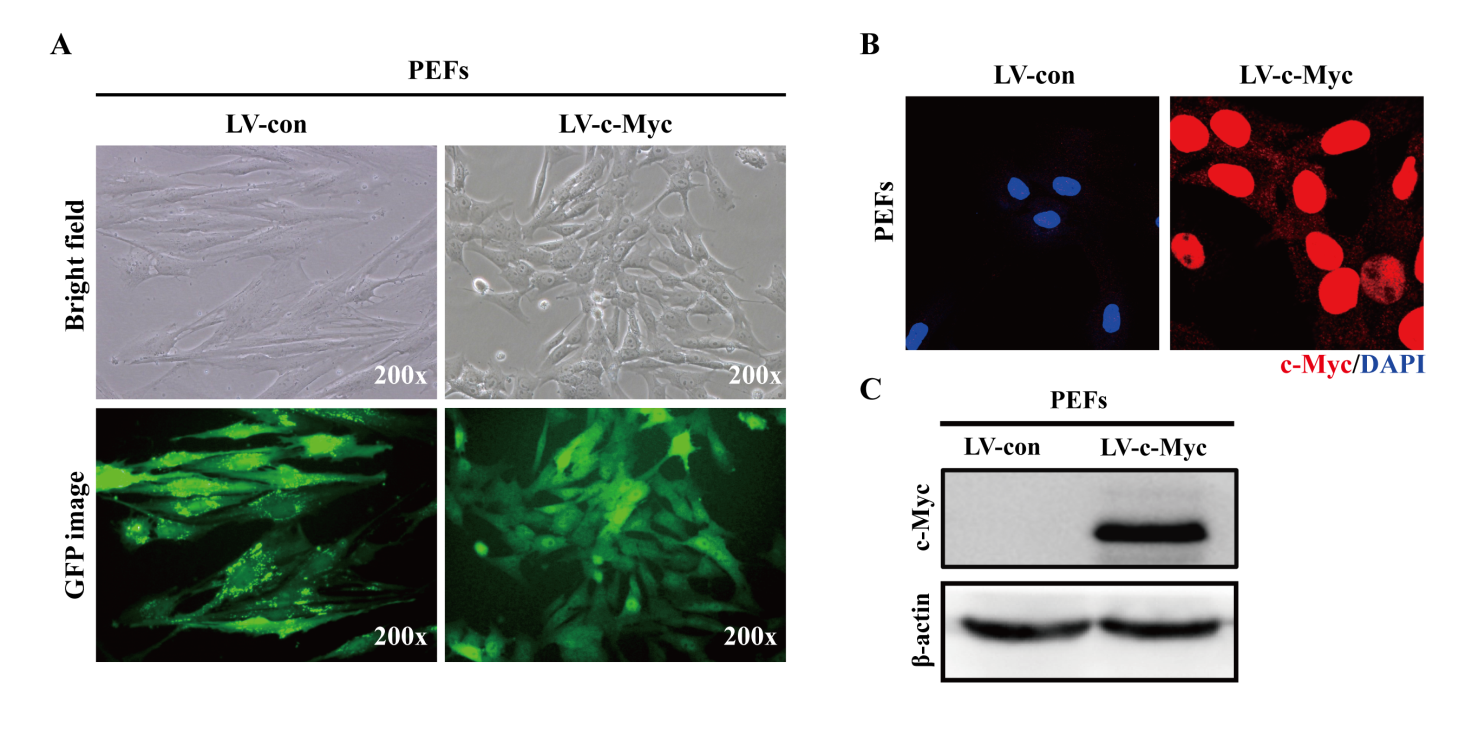
**

**Fig. S2 Generation of stable PEFs expressing EGFP and c-Myc transgenes****.**

(**A**) EGFP assay under inverted fluorescence microscopy for EGFP expression in PEFs harboring EGFP transgene.

(**B** and **C**) The detection of c-Myc expression in PEFs carrying c-Myc transgene by immunofluorescence (B) and Western blot (C).


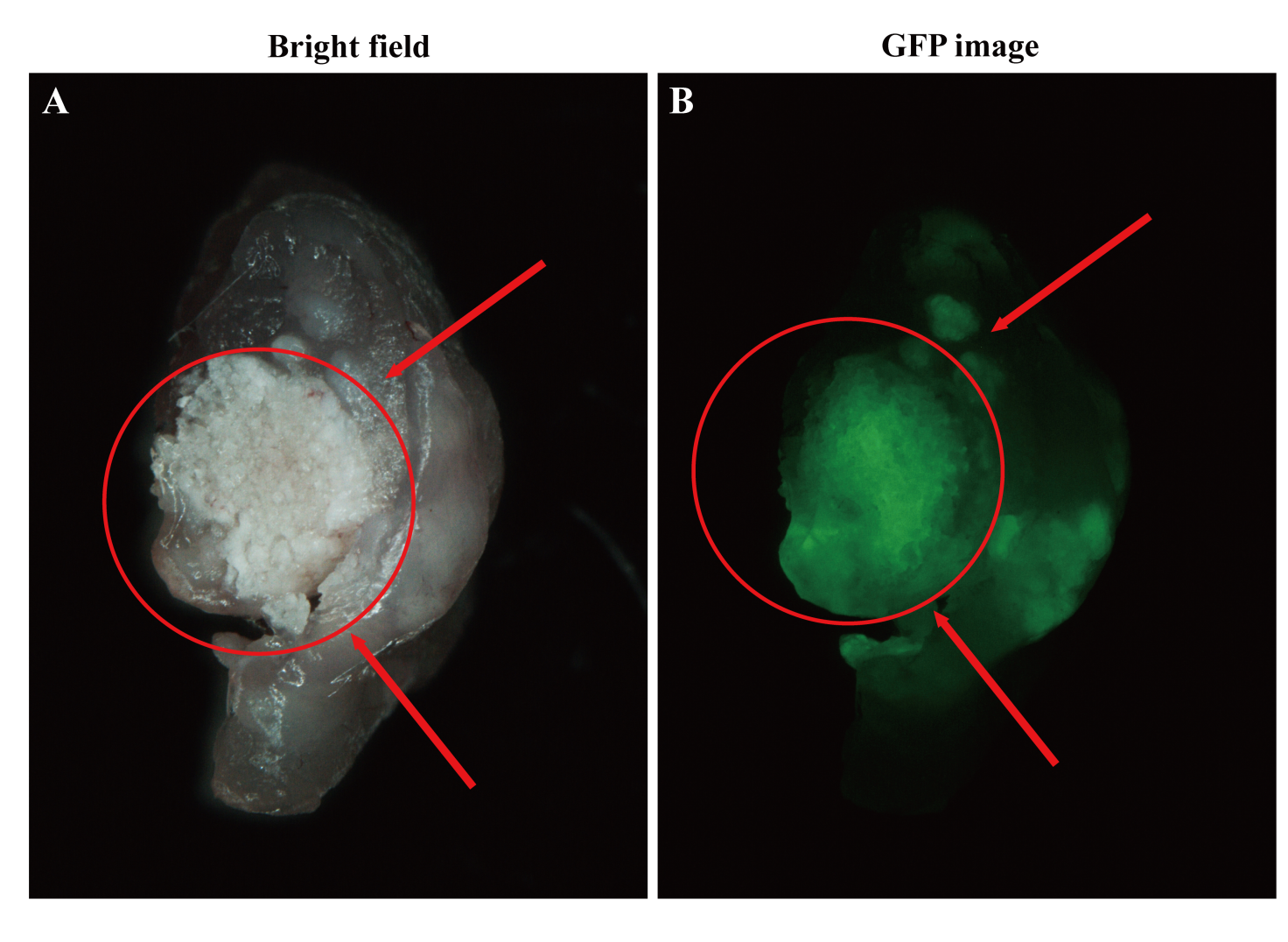


**Fig. S3 Imaging of the cross section of the suspected cartilage-like tissues developed from the transplanted c-Myc-expressing PEFs at injected site (right) of nude mouse (801)**(shown in Figure 2B-a,b,c)**.**

(**A**) Picture of the cross section of GFP-positive graft (harboring LV-c-Myc)[shown in Figure 2B-b,c; right)].

(**B**) GFP assay for the cross section[shown in (A)]of GFP-positive graft[shown in Figure 2B-b,c; right)].


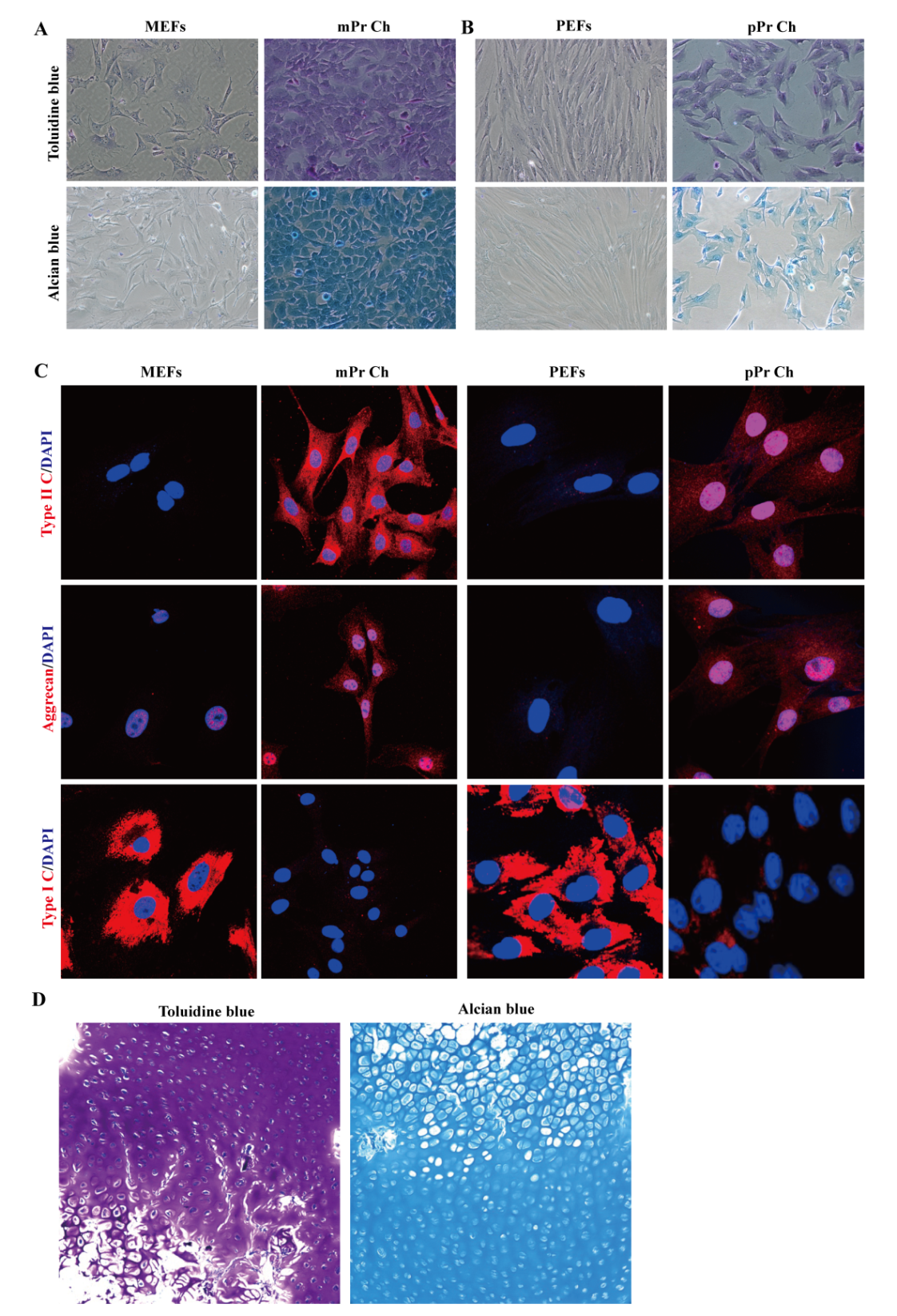


**Fig. S4 Setting up the identification system of mouse and pig chondrocytes.**

(**A** and **B**) Toluidine blue staining and alcian blue staining for mPr Ch(A) and pPr Ch(B).

#### (C) [Immunofluorescence](http://www.so.com/link?m=aleralRB6rFEvioxj5DKc%2BPZBt4vUO6Js7F%2Bj7sQGtE%2B65r1b5cJ6Qnr8ACR0YtOWrt%2BZNlSu5VOvSy29erKMeyZcScPuKr3P0V6TT%2FE5eQaxSgMYKIzqu4WD5lulhNgk4UV3BPLoEXmA2WCq6FymeIpNingt5JbjtRl%2BjSNCro5Q6IVFGAjpos2M5qJ3vBZ6rXuiDw%3D%3D) (IF) assay for the indicated protein expression in mPr Ch and pPr Ch.

Type I C: type I collagen; type II C: type II collagen.

(**D**) Toluidine blue staining and alcian blue staining for porcine cartilage tissue.


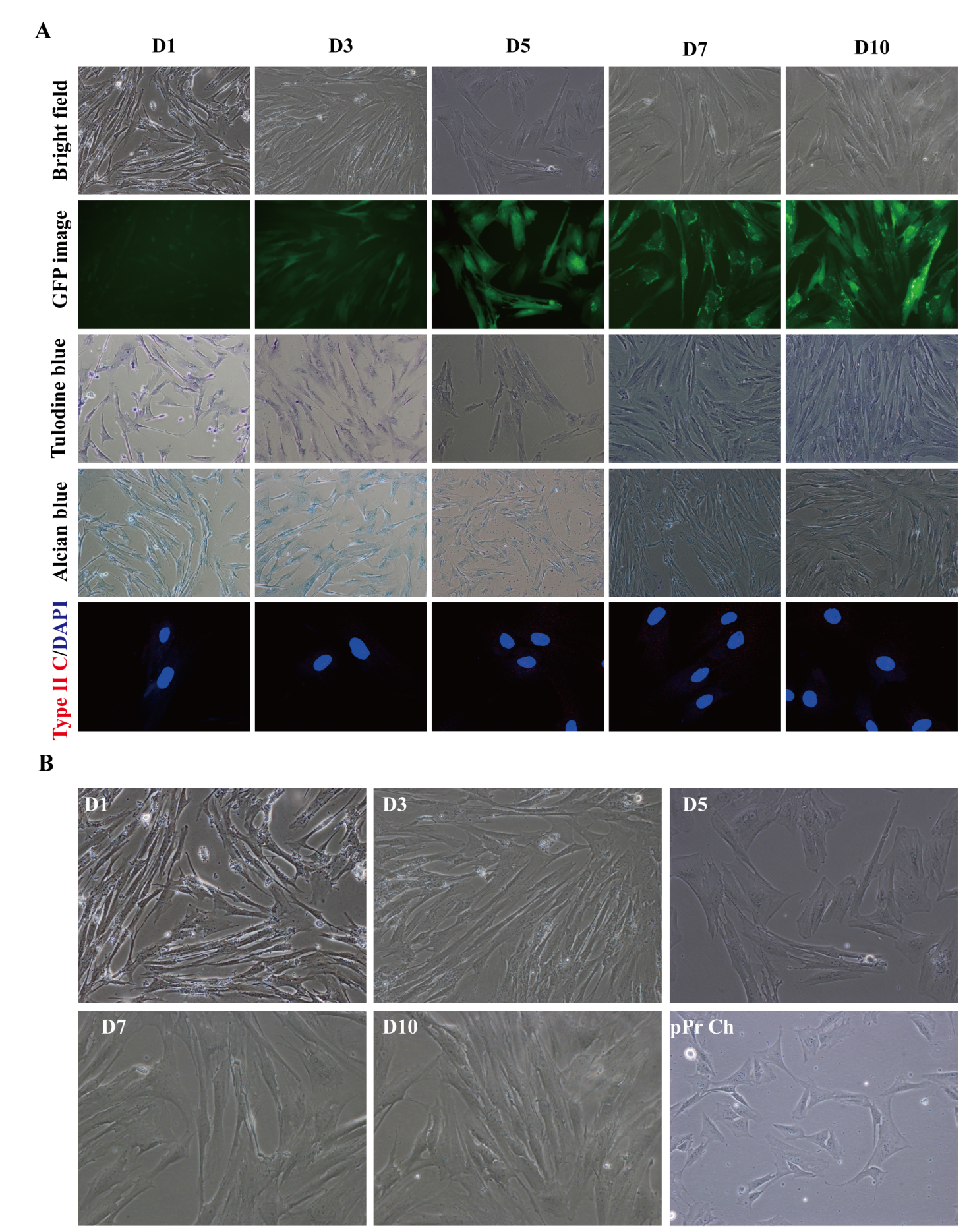


**Fig. S5 The changes in vector-expressing PEFs from D1 to D10.**

(**A**) The potential changes assessed by cell morphology, special staining and type II collagen (type II C) expression.

(**B**) Cell morphological changes of vector-expressing PEFs from D1 to D10.

**
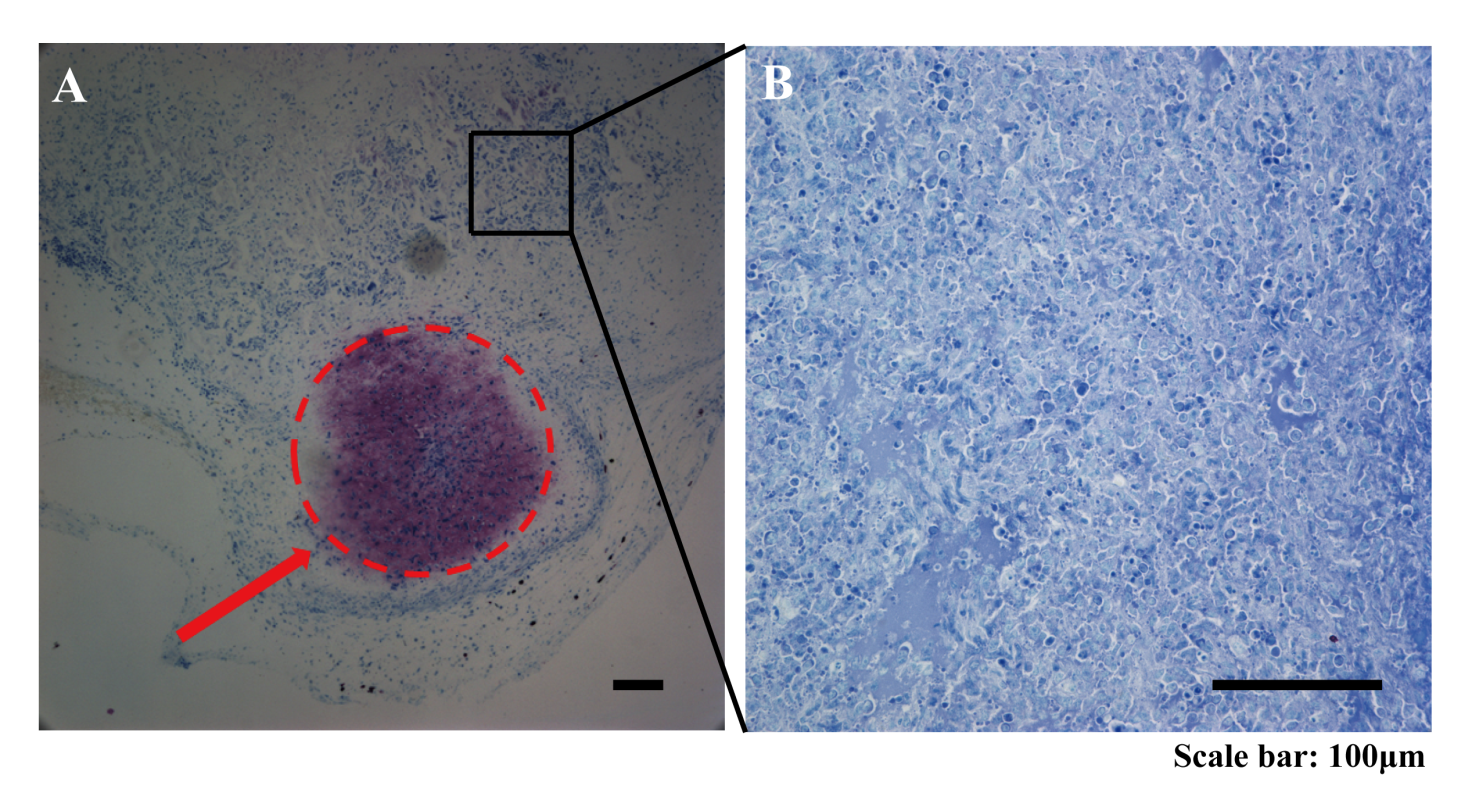
**

**Fig. S6 Injection of piCLCs produced cartilaginous tissues and tumorous tissues.**

**(A)** Toluidine blue staining of tissue section of tumorous portion and cartilaginous portion (circled region #2, shown in Figure 7E-b).

The cartilaginous portion (shown in the circle marked with red-dotted line), as indicated by intense toluidine blue staining, was surrounded by large tumors.

**(B)** High magnification view of tumorous portion.

(B) is higher magnifications of the boxed region indicated in (A).

**Supplemental Tables**

**Table S1 Results of subcutaneous injection of piCLCs into nude mice**

| **Sacrifice after injection (weeks)** | **Injected**  **cell**  **lines** | **Number of** **injected**  **sites** | **Number of sites with tissue**^1^ | **Number of sites with tumor**^2^ |
| --- | --- | --- | --- | --- |
| 4 | piCLCs | 6 | 6 | 0 |
| 8 | piCLCs | 16 | 15 | 0 |
| 12 | piCLCs | 16 | 16 | 2 |
| 16 | piCLCs | 10 | 10 | 4 |
| 12 | Vector-expressing PEFs | 10 | 0 | 0 |

^1^Number of sites where tissue were histologically recognized.

^2^Number of sites where tumors were histologically recognized.

**Table S2 Primers for qRT-PCR analysis of pig cartilage-related gene expression**

| **Gene** | **Forward primer (5’-3’)** | **Reverse primer (5’-3’)** |
| --- | --- | --- |
| Co2a1 | TCAGCGTCAGACCTACCT | GATATTCCCTCCACCACTA |
| Col1a1 | GAAGAAGACATCCCACCAGTCA | CAGATCACGTCATCGCACAA |
| Col1a2 | ACCGTTCTTCACGATACTTG | TCTCGGCTCGCTTTACAC |
| Aggrecan | ACAGACCCAGTAACCAATG | CTGAGCAGGGATGAAGAT |
| Sox5 | GGCACAAGATTCGGGAGG | ATGGGAAACCGTGTAGGG |
| Sox6 | AGTCTGGTTGGAGGTTACGG | GGAGGCAAACTTATTGTTGAG |
| Sox9 | CACGAGGAGGAAGTAAAGG | TGAGGTCCTGAGGTTTGG |
| Kif4 | CCATCAAGCAGCCCAAACCT | CAGGCAGGTCAGTTGGTTCT |
| GAPDH | TTGGCTACAGCAACAGGG | CTGGGATGGAAACTGGAAGT |

**Table S3 List of antibodies and suppliers used for immunofluorescence, immunohistochemistry and Western blot**

| **Antibody** | **Isotype** | **Suppliers** |
| --- | --- | --- |
| Type I collagen | Rabbit polyclonal | Abcam |
| Type II collagen | mouse monoclonal | Thermo |
| Aggrecan | Rabbit polyclonal | Santa Cruz |
| GFP | mouse monoclonal | Bioworld Technology |
| c-Myc | Rabbit monoclonal | Abcam |
| β-actin | Rabbit | Cell Signaling |
